# Development and characterization of three cell culture systems to investigate the relationship between primary bone marrow adipocytes and myeloma cells

**DOI:** 10.3389/fonc.2022.912834

**Published:** 2023-01-11

**Authors:** Heather Fairfield, Rebecca Condruti, Mariah Farrell, Reagan Di Iorio, Carlos A. Gartner, Calvin Vary, Michaela R. Reagan

**Affiliations:** ^1^ MaineHealth Institute for Research, Scarborough, ME, United States; ^2^ University of Maine Graduate School of Biomedical Science and Engineering, University of Maine, Orono, ME, United States; ^3^ Tufts University School of Medicine, Boston, MA, United States; ^4^ University of New England, Biddeford, ME, United States

**Keywords:** myeloma, bone marrow adipose, 3D, silk scaffolds, tissue engineering, adipocytes

## Abstract

The unique properties of the bone marrow (BM) allow for migration and proliferation of multiple myeloma (MM) cells while also providing the perfect environment for development of quiescent, drug-resistant MM cell clones. BM adipocytes (BMAds) have recently been identified as important contributors to systemic adipokine levels, bone strength, hematopoiesis, and progression of metastatic and primary BM cancers, such as MM. Recent studies in myeloma suggest that BMAds can be reprogrammed by tumor cells to contribute to myeloma-induced bone disease, and, reciprocally, BMAds support MM cells *in vitro*. Importantly, most data investigating BMAds have been generated using adipocytes generated by differentiating BM-derived mesenchymal stromal cells (BMSCs) into adipocytes *in vitro* using adipogenic media, due to the extreme technical challenges associated with isolating and culturing primary adipocytes. However, if studies could be performed with primary adipocytes, then they likely will recapitulate *in vivo* biology better than BMSC-derived adipocytes, as the differentiation process is artificial and differs from *in vivo* differentiation, and progenitor cell(s) of the primary BMAd (pBMAds) may not be the same as the BMSCs precursors used for adipogenic differentiation *in vitro*. Therefore, we developed and refined three methods for culturing pBMAds: two-dimensional (2D) coverslips, 2D transwells, and three-dimensional (3D) silk scaffolds, all of which can be cultured alone or with MM cells to investigate bidirectional tumor-host signaling. To develop an *in vitro* model with a tissue-like structure to mimic the BM microenvironment, we developed the first 3D, tissue engineered model utilizing pBMAds derived from human BM. We found that pBMAds, which are extremely fragile, can be isolated and stably cultured in 2D for 10 days and in 3D for up to 4 week *in vitro*. To investigate the relationship between pBMAds and myeloma, MM cells can be added to investigate physical relationships through confocal imaging and soluble signaling molecules *via* mass spectrometry. In summary, we developed three *in vitro* cell culture systems to study pBMAds and myeloma cells, which could be adapted to investigate many diseases and biological processes involving the BM, including other bone-homing tumor types.

## Introduction

Multiple myeloma (MM) is a malignant B cell neoplasm characterized by uncontrolled growth of mutated plasma cells within the bone marrow (BM) (1, 2). Overt (symptomatic) myeloma results in BM infiltration and a disconnect in the normal equilibrium between osteoblastic (bone building) and osteoclastic (bone breakdown and resorption) activities with a skew toward net bone loss. The unique properties of the BM allow for migration and proliferation of MM cells while also providing a supportive environment for evolution of quiescent, drug-resistant cells (3). Recently, therapies that target the marrow microenvironment rather than the tumor cells directly have proven to be effective in inhibiting tumor growth and osteolysis (4–6). In addition to osteoblasts and osteoclasts, other cells in the BM niche affect MM progression, including osteocytes (7), BM-derived mesenchymal stromal cells (BMSCs) (8), and BM adipocytes (BMAds), as we and others have shown (9–11).

BMAds are important contributors to systemic adipokine levels (12), as well as regulators of bone (13) *via* RANKL expression (14) and hematopoiesis *via* stem cell factor (SCF) production (15), and have recently been implicated in bone metastatic cancers such as acute myeloid leukemia (16) and breast cancer (17). Interestingly, BM adipose tissue (BMAT) expands with aging and obesity (18, 19), which are the two key risk factors for MM (20–22), suggesting that BMAds interact with and influence myeloma cells in the marrow. Indeed, adipocyte-derived factors such as monocyte chemoattractant protein 1 (MCP-1) and stromal cell-derived factor 1 (SDF1) are known chemotactic factors for myeloma cells (9, 23), whereas other factors promote myeloma proliferation (e.g., leptin) (10) and resistance to chemotherapies (e.g., leptin and adipsin) (11). Although these effects of physiologically normal adipocytes on myeloma cells have begun to be characterized, how cancer-associated adipocytes differ from healthy adipocytes (17, 24, 25) and whether these changes can be tied to disease progression is poorly understood.

Here, we built on our prior work examining the effects of myeloma cells on BMAds derived from BMSCs (26–28) to develop techniques to isolate, culture, and characterize primary BMAds (pBMAds). Our work was motivated by the knowledge that cells differentiated *in vitro* are not identical to the terminally differentiated cell type isolated directly from the body. This can be due to the potential effects of differentiation media, artificial/two-dimensional (2D)/monoculture conditions devoid of the correct microenvironmental signals or the incorrect progenitor cell being used based on assumptions or technical difficulties in obtaining or determining the true *in vivo* progenitor cell (29, 30). Although the analysis of pBMAds isolated and processed immediately from BM has been described (31, 32), protocols for the collection of pBMAds are still relatively novel, and, as such, very few studies utilizing cell culture and experimentation with pBMAds *in vitro* have been described previously. Indeed, prior publications outline experiments utilizing pBMAds *in vitro* in which pBMAds were grown in culture medium–filled flasks for “ceiling” or “floating” cultures (33–35), in transwells (34), or on coverslips (36), but only one study performed pBMAd co-culture with tumor cells (37), and none performed this in a three-dimensional (3D) system.

Thus, we further developed and utilized three methods of *in vitro* culture of pBMAds [using 2D ceiling coverslips, 2D ceiling transwells, and 3D silk scaffolds (27, 38)], which can be used for mono- or co-cultures with MM or other tumor cells to investigate bidirectional tumor-host signaling. Many findings demonstrating that, using 3D cultures and matching material substrate, more closely resembling the mechanical and physical properties seen *in vivo*, improves accuracy in modeling tumors *in vitro* (39–41). Thus, we developed an *in vitro* model with a 3D, tissue-like structure to mimic the BM microenvironment, which represents the first 3D model utilizing human pBMAds. Using the methods that we developed, we found that the fragile pBMAds can be isolated and stably cultured in 2D for 2 weeks and in 3D for up to 4 weeks *in vitro*. To investigate the relationship between pBMAds and myeloma disease progression, MM cells were added to the cultures and analyzed for cell phenotype and localization, using confocal imaging, and the effects on soluble signaling molecules *via* mass spectrometry. In summary, we developed three *in vitro* cell culture systems to study pBMAds and myeloma cells, which could be adapted to investigate many diseases and biological processes involving BMAds.

## Materials and equipment

### Materials and reagents

*Note: All materials and reagents are stored at room temperature unless otherwise noted.*

1. Lithium heparin VACUETTE tubes, 9 ml (Greiner Bio-One, Cat. no. 455084)
2. MM.1S or RPMI-8226 human myeloma cells
3. Dulbecco’s modified Eagle medium/F12 (DMEM/F12; Corning, Cat. no. 10-090-CM), stored at 4°C
4. Premium-grade fetal bovine serum (FBS; Seradigm, Cat. no. 1500-500), stored at −20°C
5. Penicillin/streptomycin (pen/strep; VWR, Cat. no. 97063-708), stored at −20°C
6. Antibiotic-antimycotic (anti-anti; VWR, Cat. no. 45000-616), stored at −20°C
7. RPMI-1640 (VWR, cat. no. 45000-396), stored at 4°C
8. Trypan blue (GE Healthcare, cat. no. SV30084.01)
9. Six-well plates for tissue culture (VWR, Cat. no. 734-2323)
10. Phosphate-buffered saline (PBS; 1×) (VWR, Cat. no. 97062-340)
11. Blood collection tubes with ethylenediaminetetraacetic acid (EDTA) (BD Biosciences, Vacutainer EDTAK2, Cat. no. BD367525)
12. Distilled water (dH_2_O)
13. Ninety-six–well plates with round bottom (Sigma-Aldrich, Cat. no. M9436-100EA)
14. T75 flasks (VWR, Cat. no. 10062-860)
15. Falcon tubes (15 ml) (VWR, Cat. no. 525-0604)
16. Falcon tubes (50 ml) (VWR, Cat. no. 525-0610)
17. Microtubes (1.5 ml) (Enzifarma, Cat. no. P10202)
18. Cryo vials (VWR, Cat. no. 89094-810)
19. Transwell membranes (0.4 µm) (Corning, Cat. no. 353090)
20. Rectangular cover glass, 50 × 24 mm, #2 (VWR, Cat. no. 48382-136)
21. QIAzol (Qiagen, Cat. no. 79306)
22. Silk scaffolds [provided by Reagan lab, or made as described (27)], autoclaved and stored in sterile H_2_O at 4°C.
23. Optional: Scrapers, 11-mm blade (USA Scientific, Cat. no. CC7600-0220)
24. Optional: White bioluminescent imaging (BLI) plates (CELLSTAR, Cat. no. 655083)
25. Optional: CellTiter-Glo (Promega, Cat. no. G9241), stored at −20°C.
26. Optional: RealTime-Glo (Promega, Cat. no. JA1011), stored at −20°C.
27. Optional: Luciferin [IVISbrite D-Luciferin Potassium Salt Bioluminescent Substrate (1 g) (XenoLight); PerkinElmer, Cat. no. 122799]; see below for recipes; powder and reconstituted solution both stored at −20°C.
28. Optional: Qiagen miRNeasy Kit (Qiagen, Cat. no. 217084)
29. Optional: RNase-Free DNase Set (Qiagen, Cat. no. 79254)
30. Optional: Neutral Buffered Formalin (VWR, Cat. no. 89370-094)
31. Optional: Triton X-100 (Sigma-Aldrich, CAS no. 9036-19-5)
32. Optional: DAPI (Life Technologies, Cat. no. D1306), stored at −20°C
33. Optional: Phalloidin (Life Technologies, Cat. no. A12379, stored at −20°C
34. Optional: Methanol (BDH Chemicals, Cat. no. BDH1135-1LP)
35. Optional: Oil Red O (VWR, Cat. no. 97062-192)
36. Optional: Isopropanol (BDH Chemicals, Cat. no. BDH1133-4LP, CAS no. 67-63-0)
37. Optional: 0.2-µm filter
38. Optional: Glass bottom dishes (1.5-mm uncoated, gamma-irradiated; MatTek, Cat. no. P50G-2-14-F-GRID, or similar)
39. Optional: Reagents for mass spectrometry:

- Ethanol (stored at −20°C)
- Tris
- Urea
- Bicinchoninic acid (BCA) assay (Thermo Scientific Pierce, Waltham, MA)
- TCEP [tris(2-carboxyethyl)phosphine hydrochloride (Strem Chemicals, Newburyport, MA]
- Iodoacetamide (Proteomics grade, Thermo Scientific Pierce)
- 2-Mercaptoethanol (Thermo Scientific Pierce)
- Calcium chloride, dihydrate (OmniPur, Sigma-Aldrich, St. Louis, MO)
- Sequencing-grade modified trypsin (Promega, Madison, WI)
- Acetonitrile (LC-MS-grade, Honeywell Burdick & Jackson, Muskegon, MI)
- Formic acid (Optima grade, Fisher Scientific, Waltham, MA)
- Top Tip Micro-spin columns packed with C18 media (Glygen Corporation, Columbia, MD)
- Reverse-phase nano HPLC columns (Acclaim PepMap 100 C18, 75 µm × 150 mm, 3-µm particle, 120-Å pore)
- Water (LC-MS-grade, Honeywell Burdick & Jackson, Muskegon, MI)

### Equipment

1. Micropipettes
2. Thin forceps
3. Multichannel pipettes
4. Centrifuge
5. Biosafety cabinet with vertical flow
6. 37°C, 5% CO_2_ humidified cell culture incubator
7. Water bath able to reach 37°C
8. Hemocytometer
9. GLOMAX microplate reader (Promega, Cat. no. GM3000) (read on bioluminescence setting with standard integration time)
10. EVOS M5000 Digital Inverted Fluorescence and Transmitted Light Imaging System (or other fluorescent microscope with red, green, and blue channels) or Confocal Microscope (Leica SP5X laser scanning confocal microscope (Leica Microsystems, Buffalo Grove, IL)
11. Aluminum heat/cooling block capable of holding temperatures from −20°C to 55°C
12. Refrigerated (4°C) tabletop centrifuge
13. Centrifugal vacuum concentrator
14. TripleTOF 6600+ quadrupole time-of-flight (QTOF) mass spectrometer (Sciex, Framingham, MA) with silica capillary emitter (SilicaTip, 20 µm ID, 10 µm tip ID, New Objective, Littleton, MA)
15. Eksigent NanoLC 425 nano-UPLC Liquid Chromatography System

### Software

1. GraphPad Prism v.7
2. Leica Application Suite Advanced Fluorescence (LAS AF) Lite software or Leica LAS acquisition software
3. Sciex MarkerView software (version 1.3.1, Sciex LLC, Framingham, MA)
4. Sciex Protein Pilot software (version 5.0.2, Sciex LLC, Framingham, MA)
5. Sciex PeakView software (version 2.2, Sciex LLC, Framingham, MA)
6. STRING: Functional protein association networks; found at https://string-db.org/
7. Sciex Analyst software (version 1.7, Sciex LLC, Framingham, MA)

### Recipes

1. Supplemented DMEM/F12
  a. Add 10% FBS and 1% of anti-anti to total DMEM/F12
2. Supplemented RPMI-1640
  a. Add 10% FBS and 1% of pen/strep to total RPMI
3. Luciferin: Stock solution at 7.5 mg/ml
  a. Combine 1 g of D-luciferin powder with 133.3 ml of sterile Dulbecco’s PBS without Mg^2+^ or Ca^2+^
  b. Sterilize using a 0.22-µm filter (Corning Life Sciences)
  c. Aliquot and freeze at −20°C
4. 0.2% Triton X-100
  a. Cut end of pipette tip of P200 to transfer viscous Triton X-100 to PBS as outlined below.
  b. Add 100 µl of Triton X-100 in 50 ml of PBS and mix gently but thoroughly
5. Oil Red-O 60% solution
  a. Make stock of Oil Red-O solution by combining 0.700 g of powder in 200 ml of isopropanol
  b. Dilute to 60% solution using 1× PBS and filter (0.2 µm) to remove clumps
  c. Store at room temperature away from light
6. Live-Dead Imaging on pBMAds on Silk Scaffolds
  a. Either use this kit [Invitrogen™, LIVE/DEAD™ Cell Imaging Kit (488/570), Thermofisher, Cat. no. R37601], according to manufacturer’s instructions, OR:
  b. Make your own stock solution using the following:
    i. Calcein Stock Solution (4 mM) (to stain live cells)
      1. Dissolve 50 µg of Calcein (contents of one vial, perform this in the vial) in 12.5 µl of dimethyl sulfoxide (DMSO); keep frozen and away from light.
      2. Calcein used (Thermofisher Calcein AM, Cat. no. C3100MP); other calceins can be used if other excitation/emission colors are needed
      3. Excitation/Emission Max for Calcein AM: 494/517 nm.
      4. Note: Scaffolds have autofluorescence at many wavelengths, especially red, so making overlay maximum projection images can help distinguish dead cells from silk scaffolds.
    ii. Ethidium Homodimer (EthD-1) (2 mM) Stock Solution (to stain dead cells)
      1. Make a 1:4 DMSO/Water solution (vol:vol) (150 µl of DMSO + 600 µl of water).
      2. Dissolve 1 mg of EthD-1 into 583 µl of 1:4 DMSO/water solution.
      3. Ethidium Homodimer-1 used [Thermofisher Ethidium Homodimer-1 (EthD-1), Cat. no. E1169].
      4. Excitation/Emission Max for EthD-1: 528/617 nm.
    iii. Add 5 µl of 4 mM Calcein Stock + 20 µl of 2 mM EthD-1 into 10 ml of PBS.
    iv. Store/aliquot extra stocks at −20°C and protect from light.
  c. Scale down or up as needed: Use enough solution to cover scaffolds or samples.
  d. Add and mix solution around samples, wait 30 min, keep in the dark in incubator.
  e. Rinse gently with PBS and then image.
7. Optional: Solutions for mass spectrometry
  a. Tris buffer of 25 mM (pH 0–8.5) containing 8.0 M urea.
  b. Tris buffer of 25 mM (pH 8.0–8.5) containing 1.9 M calcium chloride.
  c. TCEP (tris[2-carboxyethyl] phosphine hydrochloride) of 300 mM prepare in 1.0 M Tris base.
  d. Acetonitrile solution (4%) containing 5% formic acid.
  e. Mobile phase A = 2% acetonitrile solution containing 0.1% formic acid.
  f. Mobile phase B = 99.9% acetonitrile containing 0.1% formic acid.

## Methods

### Establishment of myeloma-adipocyte co-cultures

#### Primary bone marrow samples

1. Cancellous bone samples derived from the acetabulum are collected from donors (men and women) after total hip arthroplasty surgeries. Bone samples (≥2 ml in size) containing both bone fragments and BM should be collected into 9-ml lithium heparin tubes.
2. Samples can be provided as deidentified samples through a Biobank or other system after IRB approval and informed consent.
3. BMAds can then be isolated from bone and other marrow cells as described below. Note: Donor variation in size, phenotype, and lipid content within samples was observed.

#### Isolation of primary bone marrow adipocytes

1. Warm supplemented DMEM/F12 to 37°C. This medium is used for the entire pBMAd isolation protocol.
2. Remove entire BM/bone sample from initial tube and place into a 50-ml conical tube.
3. Add 10–15 ml of fresh supplemented DMEM/F12 and crush the sample with a 25-ml serological pipette to release cells. Caution: periodically clear the pipette so pieces of the sample do not clog inside the pipette.
4. Add another 10–15 ml of supplemented DMEM/F12 media and continue to break up the sample until the pieces of marrow are small and mostly white.
5. Incubate the sample at 37°C for 10–15 min to allow adipocytes to float to the surface. You should see a white/yellow lipid layer beginning to form.
6. Centrifuge the entire sample for 5 min at 1,000 rpm (180*g*, relative centrifugal force (RCF)) (we utilized a Beckman Allegra 6R Refrigerated Centrifuge with a GH-3.8 swing bucket rotor). At the end of this spin, the adipocyte/lipid layer should be clearly separated from the rest of the sample at the top meniscus. Note: A sample of pBMAds can be saved for RNA by pipetting 40 µl of the floating cells directly into a cryo vial and flash-freezing in liquid nitrogen.
7. To seed pBMAd experiments/collect pBMAds (as outlined below), collect cells from the adipocyte/lipid layer at the meniscus by slowly moving the pipette along the lipid layer as the appropriate volume of cells/liquid is drawn up inside. Note: A P1000 was used when possible to avoid damage to fragile adipocytes.
  a. For co-culture experiments using six-well plates and 0.4-µm transwell inserts, dispense 2 ml of media into each well and gently add 300 µl of pBMAds per well. This volume represents approximately 500,000–1,300,000 pBMAds per well.
  b. For co-culture experiments using 24-well plates and 0.4-µm transwell inserts, dispense 0.5 ml of media into each well, add 75 µl of pBMAds per well. This volume represents approximately 160,000–330,000 pBMAds per well. Note: Slight trimming of the end of the tip of a P200 pipette will allow for gentle collection and dispensing of adipocyte liquid cultures at this volume.
  c. For co-culture phenotyping experiments using coverslips, dispense 2 ml of media into each well of a six-well plate and gently add 300 µl of pBMAds per well.
    i. Gently place coverslip on top using forceps.
  d. For co-culture experiments using 3D silk scaffolds, 100 µl of pBMAds can be used (see methods below). This volume represents approximately 220,000–450,000 pBMAds per scaffold. Saturating the scaffolds with pBMAds leads to the best results.
8. Proceed with steps outlined below for co-culture assembly.
9. Return plates to incubator; incubate samples for 7 days prior to co-culture to allow them to adhere to coverslips or scaffolds.

#### Culture of myeloma cell lines

1. Warm supplemented RPMI-1640 to 37°C.
2. Quickly thaw MM.1S and/or RPMI-8226 cryo vial (1 ml, cells suspended in typical freezing media) by placing in a water or bead bath at 37°C.
3. With a P1000 pipette, add the cell suspension to a 15-ml tube containing 9 ml of supplemented RPMI-1640 media.
4. Centrifuge at 1,000 rpm (180*g*, RCF) for 5 min to pellet cells. Remove and discard media containing DMSO.
5. Resuspend cells in 5–10 ml of fresh supplemented RPMI-1640 media.
6. Count the cells in a hemocytometer with trypan blue to exclude dead cells and resuspend the desired concentration in supplemented RPMI-1640 media. Refer to “Day 7” of co-culture assembly protocols outlined below.
7. Myeloma cells are passaged in T75 flasks with 5 million cells seeded into 15 ml of media, fed an additional 15 ml of media every other day until flasks are full (or 80% confluency is reached). Note: MM.1S and RPMI-8226 cell lines are semi-adherent and require gentle scraping to release cells prior to passaging.
8. Extra myeloma cells can be stored in 1 ml of supplemented RPMI-1640 containing 10% DMSO (5 million cells per cryo vial) in liquid nitrogen.

#### 2D co-culture assembly

**Figure 1:**
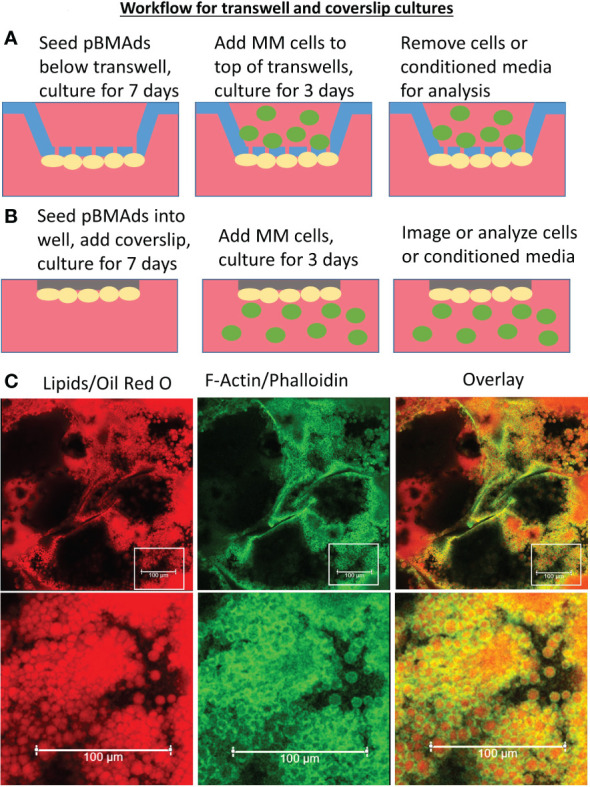
Development and characterization of 2D methods of pBMAd culture**. (A, B)** Schematics of 2D cell culture options. Green, tumor cells (floating and semi-adherent). Yellow, primary bone marrow adipocytes adherent to bottom of transwell **(A)** or to bottom of coverslip **(B)**. **(A)** Schematic of transwell culture system. **(B)** Schematic of coverslip culture system. **(C)** pBMAd donor R21-1039 imaged on cover slips after 10 days in culture. Staining with Oil Red O (red) and phalloidin (green), shown as overlay, indicates that many, but not all, lipid droplets are, in fact, adipocytes surrounded by a cytoskeleton indicated by phalloidin. Scale bars, 100 µM.

There are two methods for 2D co-culture described in this protocol: transwell co-cultures or coverslip co-cultures (**Figures 1A, B**). pBMAds grown on ceiling cultures on coverslips were used for imaging (**Figure 1C**), and transwells were used to assess effects on cell number, gene expression (qPCR), and protein content in conditioned media (CM) (using mass spec proteomics), as described below in the results section. Transwell membranes allow for soluble factor cross-talk between cells, whereas coverslip and 3D scaffolds are methods for direct co-culture that allow for cell-cell contact, and thus, the different co-culture methods also are useful for studying different aspects of the communication between tumor cells and pBMAds. This is useful to consider when designing experiments for the specific hypothesis being tested.

1. Day 0:
  a. Prepare DMEM/F12 supplemented media in advance, warm to 37°C prior to isolation of pBMAds.
  b. Gather six-well and/or 24-well plates, 0.4-µm transwell membranes, and coverslips prior to patient sample collection.
    i. Note: This transwell well size allows for soluble factor communication but not migration between the top and bottom chambers
  c. Isolate and collect pBMAds as outlined above and seed underneath transwells and/or coverslips.
    i. For transwell experiments:
      1. Add media to each empty well: six-well = 2 ml per well; 24-well = 0.5 ml per well
      2. From the adipocyte/lipid layer, pipette liquid pBMAd culture and dispense gently into each well:
        a. Six-well plates: 300 µl of pBMAd culture
        b. Twenty-four–well plates: 75 µl of pBMAd culture to each well
      3. Place transwell on top. Add media to the top of transwells:
        a. Six-well plates: 2 ml per well
        b. Twenty-four–well plates:1 ml per well
    ii. For coverslip experiments:
      1. Add 2 ml of media per well of a six-well plate
      2. Add 300 µl of pBMAd culture per well
      3. Gently place coverslip on top using forceps
  d. Return plate to incubator.
2. Days 1–6:
  a. Allow pBMAds to adhere to transwells and/or coverslips.
  b. DO NOT DISTURB culture.
3. Day 7:
  a. For transwell co-culture:
    i. Transfer transwell membranes to new wells containing fresh supplemented RPMI media (2 ml per well for six-well plates, 0.5 ml per well for 25-well plates)
    ii. Seed myeloma cells directly into the top of transwell membrane:
      1. Twenty-four–well plates: 50,000 cells per well in 1 ml of media
      2. Six-well plates: 500,000 cells per well in 2 ml of media
  b. For coverslip co-culture:
    i. Seed myeloma cells directly into new wells of a six-well plate (500,000 cells per well in 2 ml of supplemented RPMI 1640)
    ii. Gently place (i.e., float) coverslip containing adherent pBMAds on top of meniscus of media containing MM cells (or media alone), ensuring that the pBMAds are facing down
  c. Place new plates in incubator for co-culture period
4. Days 7–9: Seventy-two–hour co-culture period
5. Day 9: Harvest media and/or cells as described under “Techniques to Characterize the Co-cultures”

#### 3D co-culture assembly

**Figure 2:**
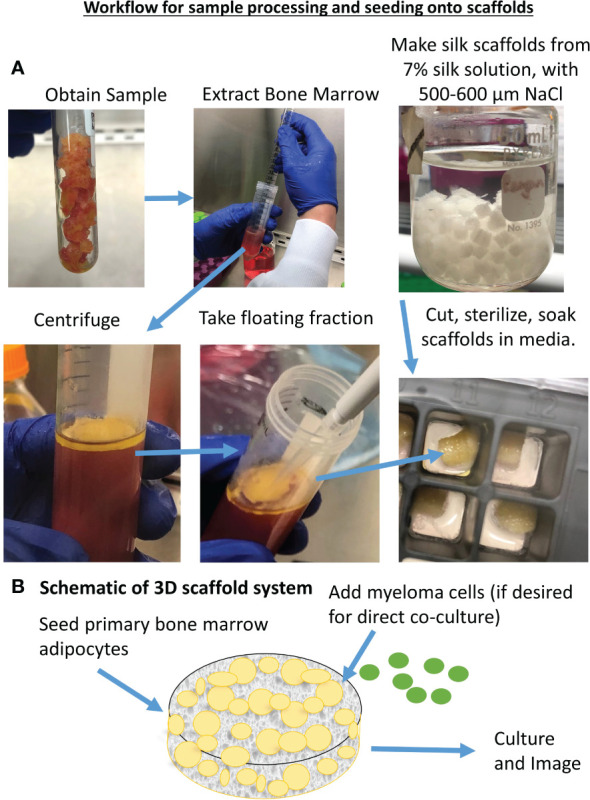
Development and characterization of 3D pBMAd culture. **(A)** Workflow for sample processing and seeding onto scaffolds. **(B)** Schematic of 3D scaffold system.

**Figure 3:**
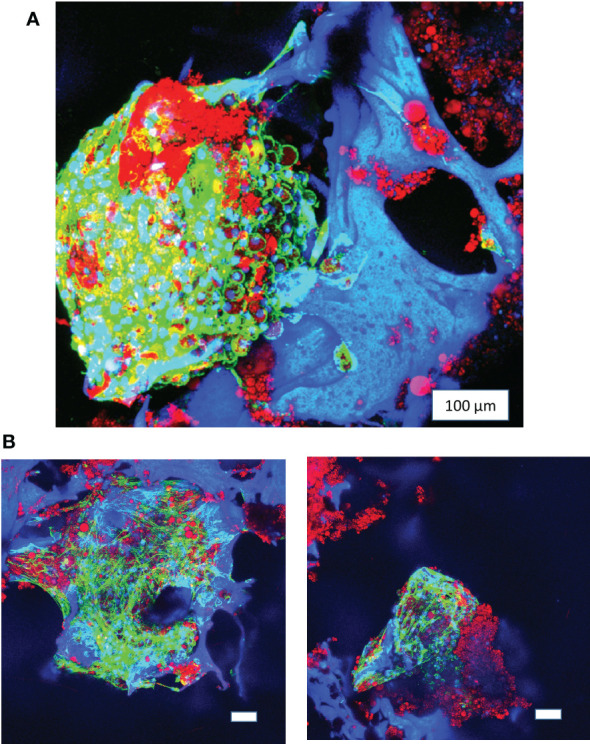
Long-term 3D culture of primary bone marrow adipocytes. **(A)** Image of pBMAds cultured for 3 weeks on silk scaffolds. **(B)** Images of pBMAds cultured for 4 weeks on silk scaffolds. All of the scale bars are 100 µm. Images are red, green, and blue overlays on confocal microscopy using maximum projection images. Blue channel is DAPI and silk scaffolds; green channel is phalloidin/F-Actin; red channel is Oil Red O staining of lipids.

**Figure 4:**
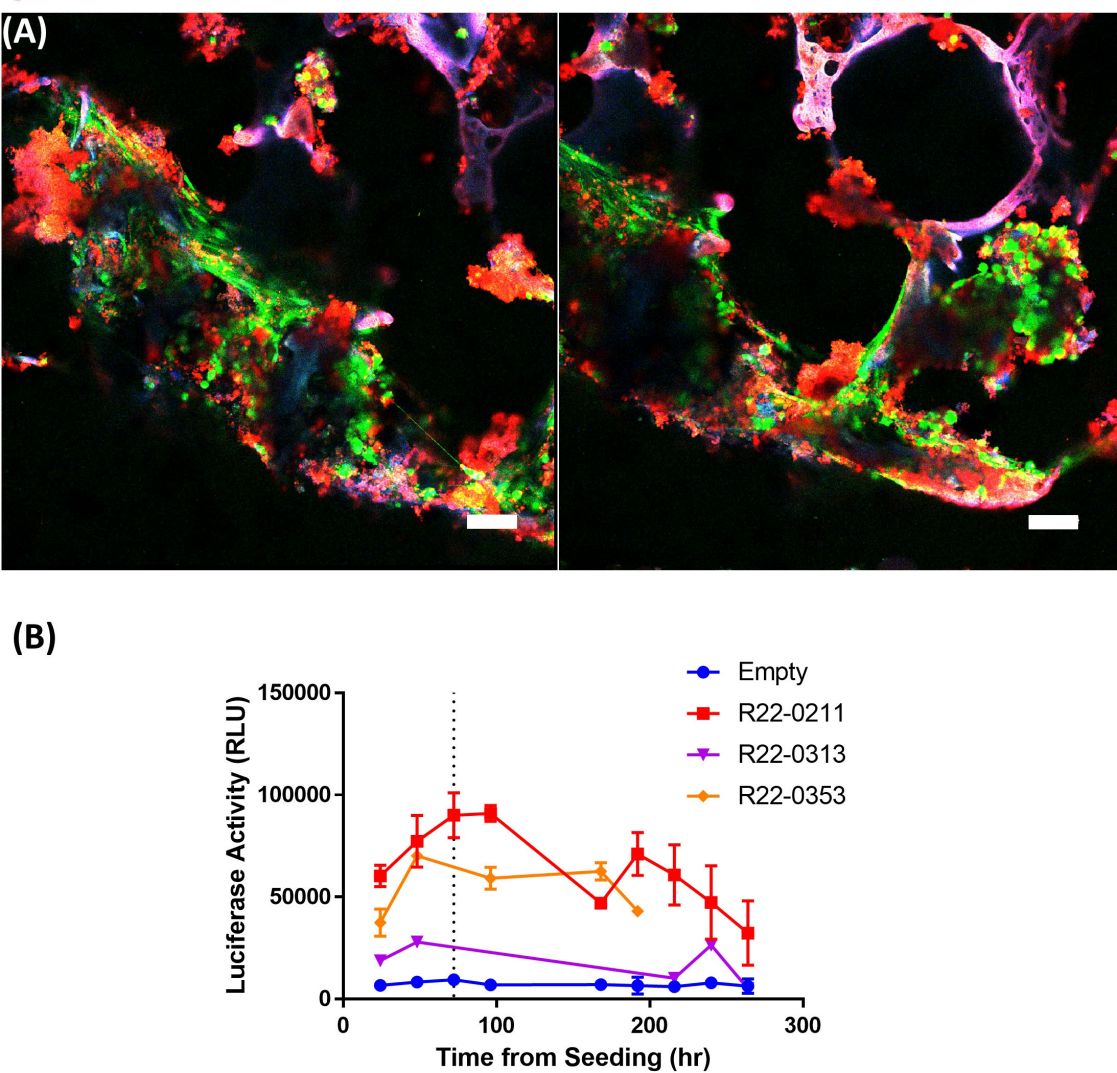
Silk scaffolds support culture of live pBMAds. Confocal maximum projection imaging of 3D scaffolds with pBMAds **(A)** after 3 weeks of culture stained with live-dead (Calcein AM, green; EthD-1; dead) stain. Lipids also appear red and scaffold autofluorescence can appear purple or pink colored. Scale bar, 100 µm. Two representative images shown. **(B)** Live pBMAds cultured on scaffolds can be quantified with RealTime-Glo (RTG). RTG reagent was seeded with adipocytes on day 0 and relative luciferase activity (RLU) measured for up to 7 days using a plate reader. RTG activity from pBMAds from three donors are quantified here, with background luciferase activity measured in empty scaffolds (n = 3); error bars in donors R22-0211 and R22-0353 represent S.E.M. of RLU from multiple scaffolds (i.e., technical replicates).

Here, we describe an original method for 3D co-culture of pBMAds and MM cells, which builds on our prior publication (27) and follows a set of steps outlined in **Figure 2**. All subsequent steps for 3D co-culture experiments used supplemented DMEM/F12 media including the addition of tumor cells that are normally cultured in supplemented RPMI-1640. We utilized the 3D co-culture system for imaging (**Figures 3**, **4A**) and characterization of CM by mass spectrometry analysis, as described below in the results section; however, future directions can include isolation of cells from direct co-culture *via* magnetically activated sorting, quantification of tumor cells by bioluminescent imaging, or quantification of gene expression in the microenvironment following RNA isolation from the entire scaffold.

1. Day 0:
  a. Silk scaffolds should be prepared and sterilized as described previously (27). Alternatively, silk scaffolds can be provided by the Reagan lab as a collaboration, or as a donation, as funds and personnel allow (contact Dr. Michaela Reagan).
    i. Punch out scaffolds using 5-mm circular biopsy punch and cut to a height of ~3 mm. Larger scaffolds can be made and utilized but should be seeded with more cells to account for the volume change.
    ii. Autoclave scaffolds either dry (wrapped in aluminum foil and placed in an autoclave pouch) or wet (in water in a glass jar with a loose lid to prevent breaking).
b. Prepare DMEM/F12 supplemented media, warm to 37^°^C prior to isolation of pBMAds
c. Prepare scaffolds prior to patient sample collection.
    i. Place a sterile scaffold into each well of a 96-well glass bottom dish.
    ii. Soak scaffolds at least overnight in culture media so that they are hydrophilic and coated with FBS-derived proteins by the time of cell seeding.
    iii. With a pipette, remove all but ~50 µl of media from scaffold so that the scaffold is moist but still capable of holding another 100-µl volume.
  d. Collect pBMAds as outlined above and seed directly on top of silk scaffold.
    i. Seed 100 µl of pBMAd culture slowly to the top and sides of each scaffold.
      1. It is an option to re-seed pBMAds that drop to the bottom of the dish back onto the scaffold to maximize the number of pBMAds seeded
    ii. Let cells incubate at 37°C for 1–2 h
    iii. Add 200 µl of media to the side of well; this volume should just cover the scaffold.
  e. Return plate to incubator.
2. Days 1–6:
  a. Allow pBMAds to adhere to silk scaffold.
  b. DO NOT DISTURB culture.
3. Day 7:
  a. Gently remove media surrounding scaffold.
  b. Seed myeloma cells directly onto scaffold: ~100,000 cells in 50–100 µl of fresh DMEM/F12 supplemented media.
  c. Let cells sit for 1–2 h prior to adding enough media to cover the scaffold (~150 µl, but not so much that spilling when transporting back to the incubator is a risk). Slowly add the media inside the well beside the scaffold to minimize disturbing the co-culture.
4. Days 7–9: Seventy-two–hour co-culture period.
5. Day 9: Harvest media and/or cells as described under “Techniques to Characterize the Co-cultures.”

#### Techniques to characterize pBMAd-MM co-cultures

**Figure 5:**
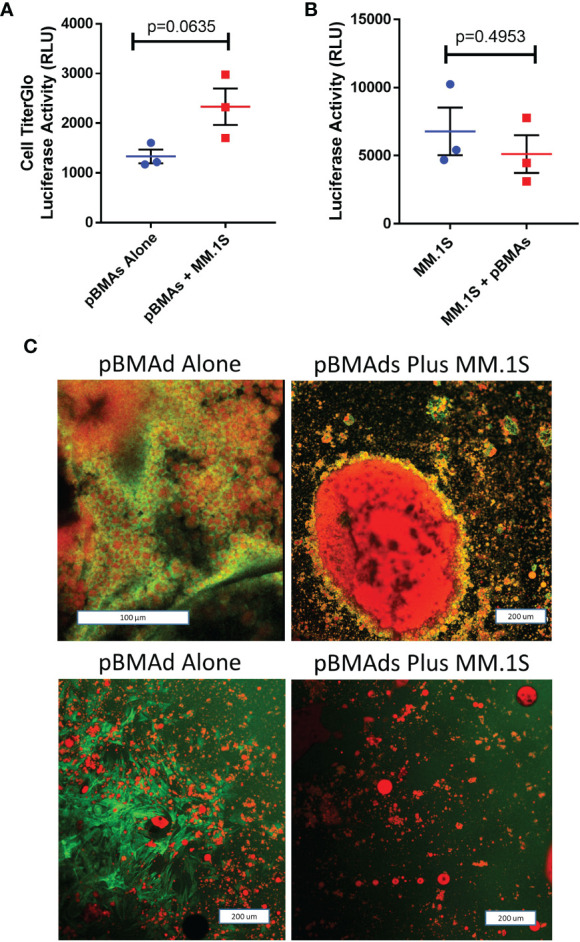
pBMAds can be co-cultured with myeloma cells in 2D transwells or coverslip cultures.**(A–C)** pBMAds were cultured on the bottom side of 24-well plate transwells for 7 days, and then, MM cells (MM.1S) were added to the tops of the transwells and cultured for 72 h. To investigate cell number in the transwell co-culture system, transwell inserts were gently lifted, medium was slowly removed from the bottom section, and the bottoms were scraped with a cell scraper to release pBMAds into the bottom of the wells. Then, a sample of the pBMAds were removed and transferred to a white plate for CellTiter-Glo analysis using a plate reader **(A)**. To assess tumor cell numbers, tumor cells in the top of the transwells were scraped gently with a pipette, and a sample was transferred to white 96-well BLI plates. Then, luciferin was spiked in and the plate was read on a plate reader; significance was investigated by t-test **(B)**. Error bars represent S.E.M. from three independent experiments. **(C)** 2D imaging of coverslips. pBMAds were seeded as ceiling cultures for 7 days, and then, tumor cells were added for 72 h. Coverslips were removed, fixed, and stained with phalloidin for F-actin (green) and Oil Red O for lipids (red) and imaged using confocal microscopy and maximum projected overlay imaging. pBMAd donor R21-1039 is on top, and R21-1114 is on bottom; left images are alone and right images are with tumor cells, as labeled. Scale bars, 200 µm.

**Figure 6:**
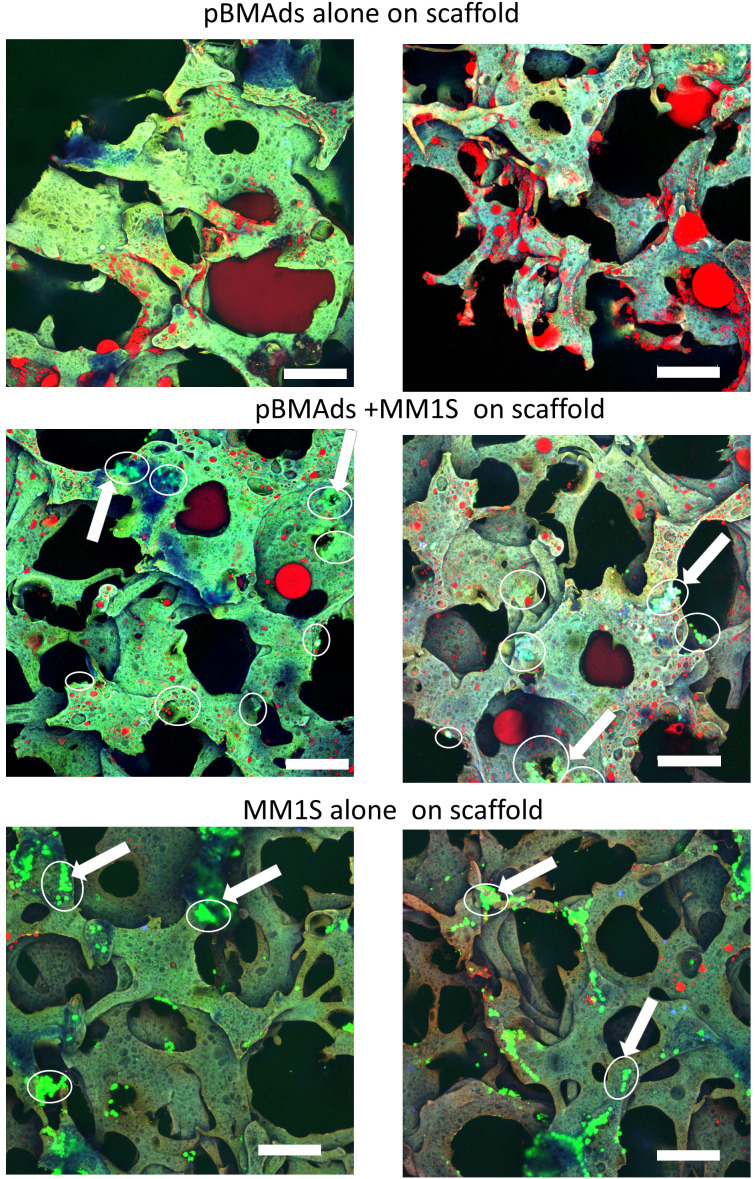
pBMAds can be co-cultured with myeloma cells in 3D silk scaffolds. pBMAds were cultured on scaffolds for 7 days, and then, medium was removed prior to the addition of either fresh media (top) or media and MM.1S cells (middle). MM.1S cells were added directly to fresh scaffolds (alone, bottom) or to pBMAd-laden scaffolds and cultured for 72 h. Imaging of scaffolds with overlay of confocal channels for GFP tumor cells (green) and lipid stain using Oil Red O (red). Scaffold autofluorescence appears light green or purple. Examples of tumor cells are indicated with arrows and circled. Scale bars, 200 µm.

**Figure 7:**
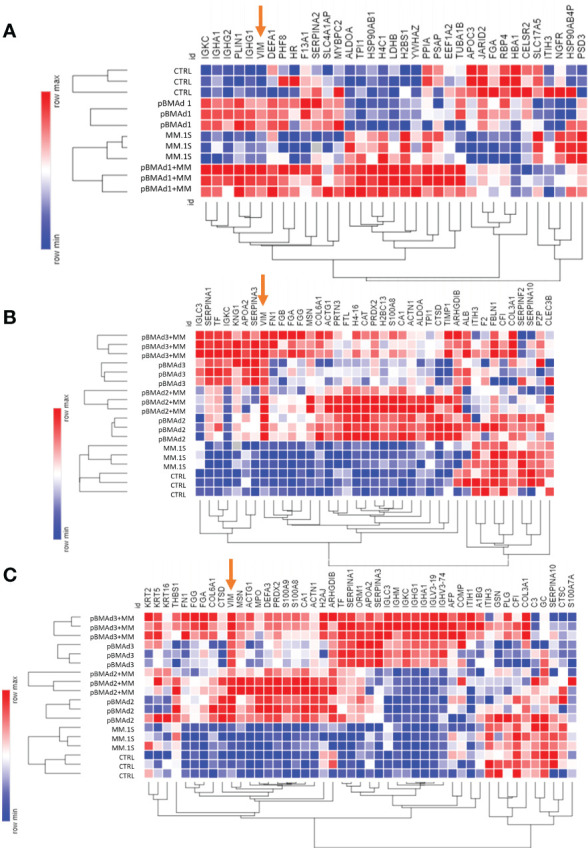
Mass spectrometry proteomic analysis of CM samples from three BMAd donors and myeloma cells grown on silk scaffolds alone and in co-culture. Differentially expressed proteins from each experiment for pBMAd donor 1 **(A)**, donor 2 **(B)**, and donor 3 are demonstrated by heatmap (Morpheus, software.broadinstitute.org/Morpheus) of log-normalized expression values; one minus Kendall correlation was used to cluster samples and proteins.

**Figure 8:**
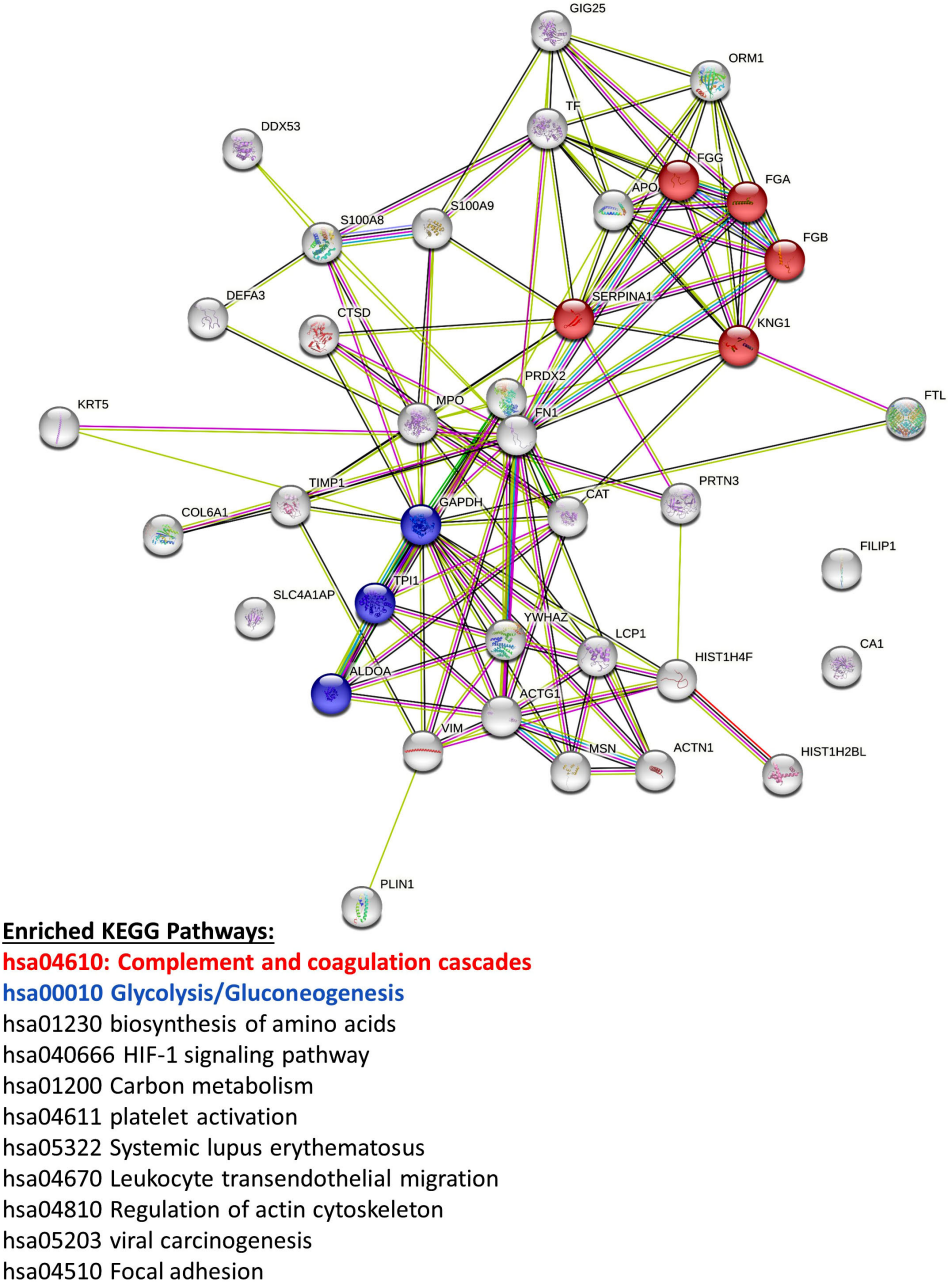
Mass spectrometry proteomic analysis of CM samples from all three pBMAd donors alone on 3D silk scaffolds. An aggregate list of differentially expressed proteins from this experiment are interconnected *via* STRING database analysis and enriched for a number of different KEGG pathways including the complement and coagulation cascades and glycolysis/gluconeogenesis.

**Figure 9:**
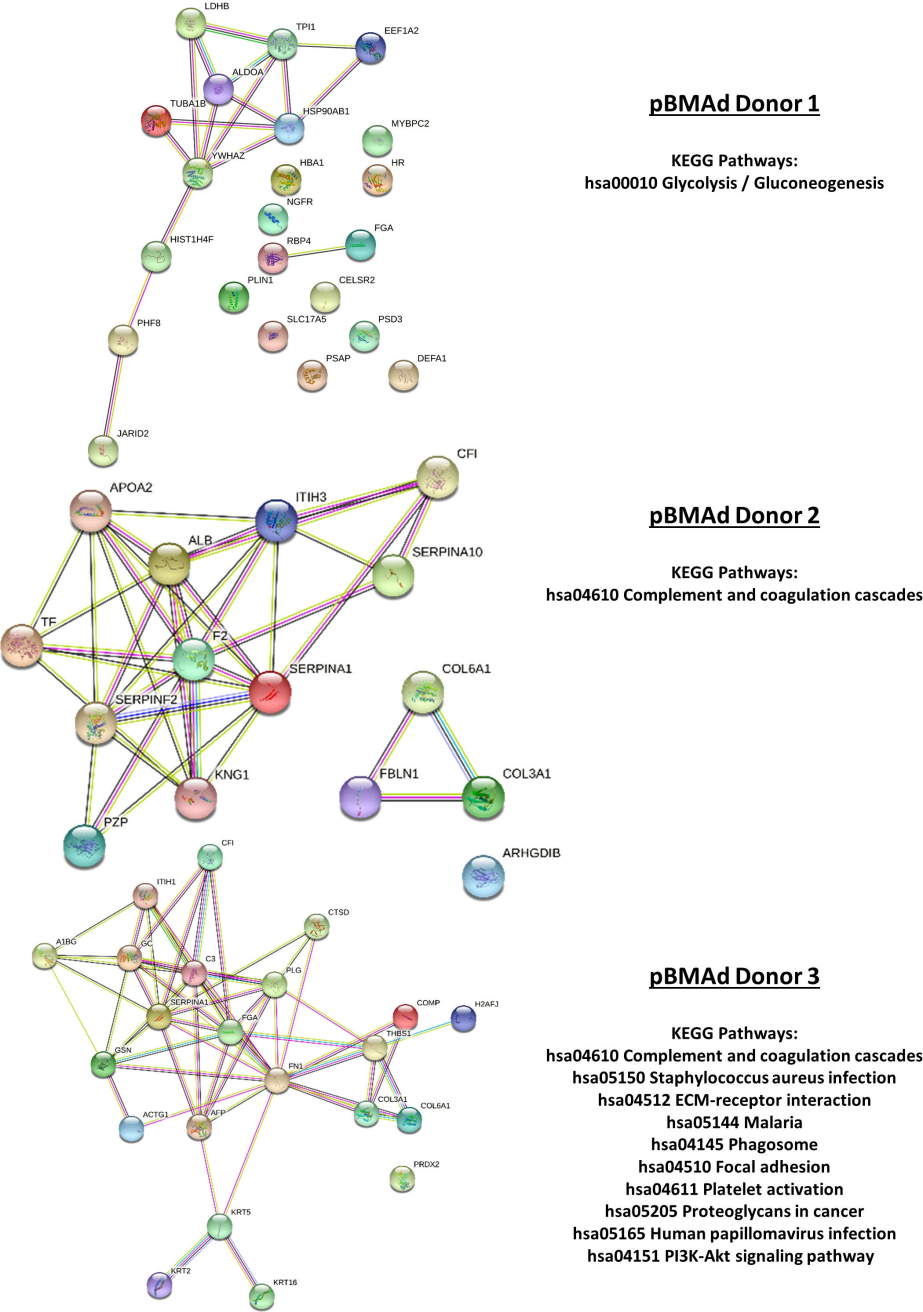
Mass spectrometry proteomic analysis of CM samples from the 3D co-culture of three pBMAd donors with myeloma cells. Differentially expressed proteins from this experiment are interconnected *via* STRING database analysis and enriched for a number of different KEGG pathways including glycolysis/gluconeogenesis, the complement and coagulation cascades, and PI3K-Akt signaling.

These co-culture systems allow for the determination of several parameters for both cell types. First, we used RealTime-Glo (**Figure 4B**) and CellTiter-Glo (**Figure 5A**) to establish viability of the pBMAds grown in culture, and exogenous luciferin to determine any potential changes in luciferase-expressing MM cell numbers in response to co-culture, as described in the results section below. To examine potential differences in gene expression, we utilized the 2D transwell co-culture system to ensure separation of the two cell types prior to RNA isolation. Next, we used imaging techniques [fluorescence (**Figure 5B**) and confocal microscopy (**Figure 6**)] to identify both cell types and determine whether these cell types were physically interacting in the 3D BM-like co-culture. To assess the soluble signals secreted by pBMAds and MM cells alone and how these signals change in co-culture systems, we utilized mass spectrometry for an unbiased, whole proteomics approach, as described in the results section below. For the experiments executed in this manuscript, media from 2D transwell co-cultures (top and bottom portions) or 3D scaffold co-cultures (**Figures 7**–**9**) were collected at day 9 (at the end of the 72-h co-culture period) for the analysis of secreted proteins (proteomics). Each cell type was also collected from either side of the transwell for RNA analysis; 2 six-well plate wells were pooled together per cell type. These methods should be adapted as needed and could be employed at any time point during co-culture. Below, days represent various points from the “co-culture assembly” protocols above.

#### Determination of cell number in 2D co-culture

1. Culture pBMAds and MM cells in 24-well transwell co-culture system for 72 h as described above.
2. On day 9 of the study (72 h of co-culture), separate myeloma cells from pBMAds to quantify cell number.
3. For myeloma cells, gently scrape cells from the top portion of the transwell with a pipette and transfer 200 µl of cell culture per well into a white BLI plate.
  a. To quantify MM.1S cells or other cells with constitutively expressed luciferase, add 10 µl of luciferin to culture in BLI plate, wait 5 min, and read on a plate reader.
  b. To quantify RPMI-8226 cells or other cells that do not express luciferase, add 100 µl of CellTiter-Glo to culture in BLI plate, wait 5 min, and read on a plate reader.
4. To collect pBMAds:
  a. Gently scrape the underside of the transwell membrane with a flat cell scraper and deposit adipocytes into media underneath transwell by dipping the membrane gently inside following scraping.
  b. Gently mix the culture by pipetting, and select pBMAd liquid culture from the same place in each well.
  c. Deposit 200 µl of pBMAds into a white BLI plate.
  d. Add 100 µl of CellTiter-Glo to pBMAds, wait 5 min, and read on a plate reader.

#### Determination of cell viability over time and collection of conditioned media in 3D scaffolds using CellTiter-Glo

For investigation of cell viability over time in our 3D scaffolds, we used two products from Promega: RealTime-Glo and CellTiter-Glo. The RealTime-Glo reagent can be added per the manufacturers’ instructions (https://www.promega.com/products/cell-health-assays/cell-viability-and-cytotoxicity-assays/realtime_glo-mt-cell-viability-assay/?catNum=G9711) at day 0 and read consecutively and reliably for up to 72 h. No additional reagents or media are required after seeding. With RealTime-Glo samples, the plate was returned to the cell culture incubator between readings instead of discarded, which allowed us to track adipocytes on individual scaffolds over time. The steps below outline a general procedure for assessment of cell viability using CellTiter-Glo, which was one method used in this manuscript and requires at least one scaffold per time point of interest as this assay is a destructive endpoint.

1. Day 0: Establish viable cells seeded at the start of each experiment. Note: Different scaffolds should be used at different times because CellTiter-Glo is a destructive endpoint.
  a. After seeding pBMAds, transfer one to two scaffolds to a white BLI plate.
  b. Add 100 µl of CellTiter-Glo directly onto scaffold and incubate 5 min at room temperature.
  c. Read BLI on a plate reader. Discard sample.
2. Day 4: Establish differences in viability from seeding to midpoint.
  a. Gently remove media surrounding one to two scaffolds and transfer scaffolds to a white BLI plate.
  b. Add 100 µl of CellTiter-Glo directly onto scaffold and incubate 5 min at room temperature.
  c. Read BLI on a plate reader. Discard sample.
3. Days 7–8: Ensure that live cells are present prior to MM co-culture.
  a. Gently remove media surrounding one to two scaffolds.
  b. Transfer scaffolds to a white BLI plate.
  c. Add 100 µl of CellTiter-Glo directly onto scaffold. Wait 5 min.
  d. Read BLI on a plate reader. Discard sample.
4. Day 9: Establish differences in viability with MM co-culture.
  a. Gently remove media surrounding scaffolds (save for proteomics or ELISA).
  b. Add 100 µl of CellTiter-Glo directly onto scaffolds. Wait 5 min.
  c. Read BLI on a plate reader.
  d. Do not forget to read scaffolds containing both pBMAds and MM cells alone for comparison.

#### Collection of conditioned media and isolation of RNA from co-cultures

1. To collect MM cells, gently scrape myeloma cells from the top portion of the transwell and collect into a pellet by centrifugation [1,000 rpm (180*g*, RCF) for 5 min].
2. Remove CM from the cell pellet and save in a new tube.
3. Resuspend myeloma cell pellet in 700 µl of QIAzol.
4. To collect pBMAds, slowly collect the CM from the six-well plate and combine with media saved above. Store CM samples at 20°C.
5. Gently scrape the underside of the transwell membrane and deposit adipocytes inside of well. Combine adipocytes from 2 six-well membranes into 700 µl of QIAzol.
6. Freeze all samples in QIAzol at −80°C.
7. RNA can be isolated utilizing Qiagen RNA extraction kits (miRNeasy with on-column DNase I treatment was used here) per the manufacturers’ instructions.

#### Imaging of pBMAds and MM cells using fluorescence and confocal microscopy

1. Fix cells in 4% neutral buffered formalin (NBF) at room temperature for 30–60 min.
  a. For coverslips, float labeled coverslips in fixative solution with forceps.
  b. For scaffolds, either remove scaffolds from the wells and place them into new wells or slowly pipette media out of the wells, and then apply ~200 µl of fixative per wells (i.e., just enough to cover scaffold).
2. Transfer to 1× PBS until ready to stain.
3. Wash the samples with PBS containing 0.2% Triton X-100.
  a. 0.5 ml per coverslip in six-well plate
  b. 300 µl per scaffold in 96-well plate
4. Stain for 1 h in staining solution away from light at room temperature with extremely gentle, slow shaking (~30 RPM).
  a. For 1 ml of solution:
    i. 200 µl of 60% Oil Red-O solution
    ii. 20 µl of DAPI (20 µg/ml)
    iii. 100 µl of phalloidin (300U/1.5 ml of methanol)
    iv. 680 µl of 0.2% Triton X-100
  b. For each coverslip, use 0.5 ml of staining solution.
  c. For each scaffold, use 300 µl of staining solution.
5. Remove staining solution and wash once with 1× PBS. Keep cells in 1× PBS for imaging.
  a. Attach coverslips to glass slides and image on a fluorescent microscope such as on an EVOS inverted fluorescence microscope.
  b.For coverslips and scaffolds, transfer these to glass bottom dishes (1.5 mm) for confocal imaging.
6. Imaging parameters
  a. Confocal imaging: 10× or 20× objective was used.
    i. Samples excited with 405-nm (diode), 488-nm (argon), and 633-nm (HeNe) lasers, with emission detected with PMT1 (blue, ~410–460 nm), PMT2 (green, ~500–590 nm) and HyD 3 (red, ~646–700 nm).
  b. Confocal hardware: Leica SP8 confocal microscope. LAS X software for data acquisition.

#### Proteomic investigation of soluble proteins secreted by pBMAds and MM cells

Proteomics can be performed on cells or CM from cultures. In our work (**Figures 7**–**9**), we analyzed the CM of cells by removing the media, centrifuging media (180*g*, RCF) to remove floating cells or debris, and transferring CM to a new tube for submission.

#### Sample preparation

1. Protein precipitation from CM samples is initiated with the addition of a 10-fold volumetric excess of ice-cold ethanol. Alternatively, a five-fold volumetric excess of acetone can be used. Samples are then placed in an aluminum block at −20°C for 1 h; then, protein pelleted in a refrigerated tabletop centrifuge (4°C) for 20 min at 16,000*g*. The overlay is removed and discarded. Protein samples are allowed to dry under ambient conditions.
2. Samples are brought to protein (1–2 μg/μl) in 25 mM Tris buffer (pH 8.0–8.5) containing 8.0 M urea. Protein content is measured relative to bovine serum albumin protein concentration standards using the BCA assay (Thermo Scientific Pierce, Waltham, MA). Approximately 100 µg of protein from each sample is used in further analysis.
3. Each sample is brought to 10 mM TCEP (Strem Chemicals, Newburyport, MA) from a 300 mM stock solution prepared in 1.0 M Tris base. Reduction of cysteine residues is performed in an aluminum heating block at 55°C for 1 h. After cooling to room temperature, each sample is brought to 25 mM iodoacetamide (Thermo Scientific Pierce, Waltham, MA) and cysteines alkylation allowed to proceed for 30 min in the dark. Reaction is quenched with the addition of 1–2 µl of 2-mercaptoethanol (Thermo Scientific, Waltham, MA).
4. Samples are diluted with 25 mM Tris buffer (pH 8.0–8.5) containing 1.0 mM calcium chloride (OmniPur, Sigma-Aldrich, St. Louis, MO) such that the urea concentration is brought below 1.0 M.
5. Trypsin stock solutions are prepared at a concentration of 2 µg/µl using the enzyme storage solution received with the trypsin. Stocks are frozen in aliquots at −80°C until needed. Sequencing-grade modified trypsin (Promega, Madison, WI) is added to a final proportion of 2% by mass relative to sample total protein as measured with the BCA assay. Proteolysis is performed overnight at 37°C in the dark.
6. Samples are evaporated to dryness using a centrifugal vacuum concentrator. Each is then brought up to protein (1–2 µg/µl) in 4% acetonitrile solution containing 5% formic acid (Optima grade, Fisher Scientific, Waltham, MA). Peptides are freed of salts and buffers using Top Tip Micro-spin columns packed with C18 media (Glygen Corporation, Columbia, MD) according to the manufacturer-suggested protocol.
7. Samples are again evaporated to dryness using a centrifugal vacuum concentrator and peptides are then brought up to protein (1–2 µg/µl) in 4% acetonitrile solution containing 5% formic acid (Optima grade)

#### LC-MS/MS

1. All sample separations performed in tandem with mass spectrometric analysis are performed on an Eksigent NanoLC 425 nano-UPLC System (Sciex, Framingham, MA) in direct-injection mode with a 3-µl sample loop.
2. Fractionation is performed on a reverse-phase nano HPLC column held at 45°C with a flow rate of 350 nl/min.
  a. Solvents are blended from LC-MS-grade water and acetonitrile.
  b. Mobile phase A is 2% acetonitrile solution, whereas mobile phase B is 99.9% acetonitrile. Both contain 0.1% formic acid.
3. Approximately 1 µg of peptides are applied to the column equilibrated at 3% B and loading continued for 12 min.
4. The sample loop is then taken out of the flow path, and the column was washed for 30 s at starting conditions.
5. A gradient to 35% B is executed at constant flow rate over 90 min followed by a 3-min gradient to 90% B.
6. The column is washed for 5 min under these conditions before being returned to starting conditions over 2 min.
7. Analysis is performed in positive mode on a TripleTOF 6600+ QTOF mass spectrometer (Sciex, Framingham, MA) running Analyst software TF 1.8.1.
  a. The column eluate is directed to the mass spectrometer source through a silica capillary emitter (SilicaTip, 20 µm ID, 10 µm tip ID, New Objective, Littleton, MA) maintained at 2,400–2,600 V.
  b. Nitrogen nebulizer gas is held at 4–6 psi, with the curtain gas at 21–25 psi. The source is kept at 150°C.
8. Data acquisition performed by information-dependent analysis (IDA) is executed under the following conditions: A parent ion scan is acquired over a range of 400–1,500 mass units using a 200-ms accumulation time. This is followed by MS/MS scans of the 50 most-intense ions detected in the parent scan over ranges from 100 to 1,500 mass units. These ions must also meet criteria of a charge state of 2^+^ to 5^+^ and of having intensities greater than a threshold of 350 counts per second (cps) to be selected for MS/MS.
  a. Accumulation times for the MS/MS scans are 20 ms.
  b. Rolling collision energies are used according to the equation recommended by the manufacturer. Collision energy spread is not used.
  c. After an ion is detected and fragmented, its mass is excluded from subsequent analysis for 15 s. Data from four to six samples run by IDA are combined and proteins identified to form a single proteome library used for SWATH results analysis (details below)
9. SWATH analysis is performed according to previously published optimized conditions tailored to the 6600+ instrument, as described (42).
  a. Briefly, SWATH MS/MS windows of variable widths are generated using a variable window calculator tool available online through Sciex (https://sciex.com/products/software).
  b. Rolling collision energies are used, as well as fragmentation conditions optimized for ions of a 2^+^ charge state.
  c. SWATH detection parameters are set to a mass range of m/z = 100–1,500 with accumulation times of 25 ms in the high-sensitivity mode.
  d. A parent-ion scan is acquired over a range of 400–1,500 mass units using a 250-ms accumulation time.
  e. Each sample is run in triplicate to obtain proper statistical parameters.

### Proteomics data analysis

1. Protein identification is performed using Protein Pilot software running the Paragon algorithm.
  a. Data are searched against a human proteome database containing over 20,000 manually annotated entries in FASTA format downloaded from the UniProt website (UP000005640). Searches were performed with cysteines modified (iodoacetamide) and with the biological modifications focus selected.
  b. A target false discovery rate of 0.05, and a thorough ID search effort is selected for any analysis. A minimum of 95% confidence is used as a threshold for peptide identification as calculated by Protein Pilot.
2. Relative quantification is performed using the SWATH processing microApp in PeakView software.
  a. Peak groups are extracted with a 99% peptide confidence threshold and 1% peptide FDR limit.
  b. SWATH chromatograms are extracted in 10-min windows with fragment ion mass tolerance set to 50 ppm.
3. Resulting protein quantitative peak areas are further analyzed using MarkerView software to compare relative quantities of all detected proteins between samples. Statistical analyses including t-tests and principal component analyses are completed for both datasets using Sciex MarkerView software. Significantly different proteins are determined *via* t-test (p < 0.05).
  a. Data are exported as Microsoft Excel files that can be analyzed.
4. Human gene symbols for overexpressed proteins can be entered into the STRING database for connectivity and enrichment analyses.

## Results

### Phenotyping pBMAds alone and in culture with myeloma cells

Utilizing adipocytes isolated from 16 different human BM donors (**Table 1**), we made both 2D (**Figure 1**) and 3D (**Figures 2**–**4**) cultures. We created 2D transwell co-cultures (**Figure 1A**) and 2D coverslip cultures (**Figures 1C, B**). Using confocal microscopy, we demonstrated unilocular lipid droplets encased by cellular membranes (defined by phalloidin staining), which appeared similar to histological sections of BM biopsies, when pBMAds were cultured using ceiling/floating cultures on coverslips (**Figure 1C**). The adipocytes adhered and survived for up to 10 days on coverslips (**Figure 1C**) and contrasted the multilocular phenotype observed in BMSCs differentiated into adipocytes *in vitro* (**Supplementary Figure 1**). In our 3D culture systems (**Figure 2**), pBMAds also consisted of unilocular lipid droplets encased by cellular membranes (**Figure 3**; **Supplementary Figure 2**). In both cases, extracellular lipids were also observed in the cultures. Of note, each donor exhibited a slightly different adipocyte phenotype in 2D and 3D cultures, and even within donors, different regions of slides or scaffolds showed variable phenotypes, demonstrating biological heterogeneity of this depot.

**Table 1:** Patient bone marrow donor information.

Donor ID Number	Figure/Supplementary Figure Number/Use	Donor Sex	Donor Age	Race	Diagnosis	BMI (kg/m^2^)
R21-0306	**Figure 5**; **Supplementary Figures 6, 7** Transwell proteomics	M	62	Caucasian	Primary osteoarthritis of right hip	30.27
R21-0400	**Figure 5**; **Supplementary Figure 6** Transwell culture	M	66	Caucasian	Degenerative joint disease left hip	25.09
R21-0460	**Figure 5**; **Supplementary Figures 6, 7** Transwell proteomics	F	70	Caucasian	Primary osteoarthritis of left hip	29.29
R21-0512	**Figure 5**; **Supplementary Figure 7** Transwell culture and coverslip	M	69	Caucasian	Primary osteoarthritis of right hip	24.74
R21-0518	**Figure 5**; **Supplementary Figures 6, 7** Transwell proteomics	F	76	Caucasian	Primary osteoarthritis of right hip	24.45
R21-0687	**Figures 3**, **4A**; **Supplementary Figure 2** Long-term 3D	F	70	Caucasian	Primary osteoarthritis of left hip	34.33
R21-0828	**Figures 7**–**9**; **Supplementary Figure 8** 3D proteomics “Donor 1”	F	79	Caucasian	Primary osteoarthritis of right hip	38.67
R21-0982	**Figure 6** 3D Scaffold Imaging	M	69	Caucasian	Primary osteoarthritis of right hip	31.14
R21-1039	**Figure 1**; **Supplementary Figures 4–6**; Coverslip and transwell culture; 3D scaffold imaging	M	61	Unknown	Primary osteoarthritis of left hip	28.66
R21-1054	**Supplementary Figure 3** 3D Scaffold Imaging	M	65	Caucasian	Primary osteoarthritis of left hip	33.46
R21-1114	**Figure 5** Transwell co-culture	F	73	Caucasian	Primary osteoarthritis of right hip	21.13
R22-0211	**Figure 4B** 3D pBMA RealTime-Glo	M	63	Caucasian	Primary osteoarthritis of left hip	27.89
R22-0313	**Figure 4B** 3D pBMA RealTime-Glo	F	19	Caucasian	Primary osteoarthritis of the left hip	24.69
R22-0353	**Figure 4B** 3D pBMA RealTime-Glo	F	63	Caucasian	Primary osteoarthritis of the right hip	40.03
R22-1189	**Figures 7**–; **Supplementary Figures 5, 8** 3D proteomics “Donor 2”	F	70	Caucasian	Primary osteoarthritis of the left hip	30.93
R22-1192	**Figures 7**–**9**; **Supplementary Figures 5, 8** 3D proteomics “Donor 3”	F	>89	Caucasian	Osteoarthritis of the right hip	33.71

In 3D, a long-term term culture was achieved up to 3 (**Figures 3A**, **4A**; **Supplementary Figure 2**) and 4 weeks (**Figure 3B**); live/dead confocal imaging showed the presence of live cells (green), as well as red clusters (which could be dead cells or autofluorescent extracellular lipids; **Figure 4A**). We also developed a second method to determine whether pBMAds were alive on scaffolds by using RealTime-Glo, a non-destructive reagent. At seeding, pBMAds from two donors (R22-0211 and R22-0353) were allowed to incubate on scaffolds for 1 h at 37°C, whereas pBMAds from another donor (R22-0313) were incubated for only 30 min at room temperature on the scaffolds, prior to adding media containing RealTime-Glo. At 24, 48, and 72 h after seeding, a stronger signal was seen from pBMAds that were allowed more time to attach to the scaffold before filling wells with media (**Figure 4B**). (Note: Seventy-two hours was the longest; RealTime-Glo was recommended for use by Promega, but it still gave signal, providing evidence of cell survival, until day 11.) These data demonstrate differences between donors and incubation times and represent a non-invasive assessment tool for pBMAds over time in 3D culture.

We next utilized our cell culture systems to investigate the relationship between pBMAds and myeloma cells. Using CellTiter-Glo for pBMAds (**Figure 5A**) and luciferin spike-in for luciferase-expressing MM.1S (**Figure 5B**), we quantified viability of cells alone or after co-culture (after removing transwells and scraping cells). We also analyzed phenotype differences in pBMAds in response to MM cells by imaging coverslips. Interestingly, not only adipocytes but also stromal cells could be observed in ceiling cultures of pBMAds, as seen in donor R21-1114 (pBMAds alone, bottom left image, **Figure 5C**). Similar images were seen with pBMAds with or without RPMI-8226 cells (**Supplementary Figure 3**). Overall, the variability in imaging of coverslips both between donors and in different fields of view within a single donor impeded our ability to quantify changes in adipocyte size or other phenotypic effects in pBMAds. With further development of the coverslip method, more quantitative data should be available using different imaging methods. We were also able to culture MM cells (white arrows indicate MM cells) with pBMAds in 3D scaffolds, as seen with donor R21-0982 (**Figure 6**) and donor R21-1039 (**Supplementary Figure 4**). Although quantification was difficult, we were able to see that pBMAds adhered to scaffolds after 7 days in culture alone, and MM cells adhered to scaffolds in pBMAd co-cultures 3 days after seeding MM.1S cells. MM.1S cells were also adherent to the scaffold alone when grown in monoculture. Viability was quantified in scaffolds for each cell type alone and in co-culture using CellTiter-Glo bioluminescence (**Supplementary Figure 5**).

### Utilizing qRT-PCR and proteomic analysis to characterize pBMAds and MM cells *in vitro*

We next explored gene and protein-level characteristics of pBMAs. RNA was successfully isolated from pBMAds from the 72-h, transwell co-culture systems, although there were challenges yield and donor variability. We predicted a suppressive effect of MM co-culture on the expression of mature adipocyte marker fatty acid binding protein 4 (*FABP4*) (**Supplementary Figure 6A**) consistent with our prior studies in BMSC-derived BMAds (26), or changes in *CXCL1* or *TGFB*, but this was not observed. Because BMSC-derived adipocytes have been shown to regulate dexamethasone resistance in myeloma (11, 26), we also investigated the expression of two genes involved in glucocorticoid receptor trafficking in MM cells previously found to be upregulated in MM cells by BMAds (*TSC22D3* and *FKBP5*); no differences in response to pBMAd co-culture relative to MM cells alone were found (**Supplementary Figure 6B**), suggesting differences between pBMAds and BMSC-derived BMAds in their relationship with myeloma cells, which warrants further study.

Next, to investigate the relationship between pBMAds and MM cells, we collected CM from pBMAds alone, MM cells alone, and the co-culture, in our 2D transwell system and performed proteomic analysis *via* mass spectrometry on these three groups. We detected a total of 293 proteins across the three sample types, which were used to perform the PCA (**Supplementary Figure 7A**), which showed distinct groupings. A total of 16 differentially expressed proteins (DEPs; defined to be significant if p < 0.05 by t-test, |log2FC|>0.17) were detected (**Supplementary Figure 7B**). These included proteins elevated in MM.1S alone, compared with pBMAds alone (PGAM4, NIPBL, AFM, KMT2B, and EEF1A2), and those elevated in pBMAds compared with MM.1S alone (SPARC) (**Supplementary Table 1**). Co-culture CM contained elevated proteins (AFP and FMOD) and decreased proteins (FABP1 and TAF4B) compared with pBMAd alone CM (**Supplementary Table 2**). Five proteins were also significantly increased, and two decreased, in the co-culture condition compared with MM.1S cells alone (**Supplementary Table 3**). MM cell-induced changes in adipocyte protein secretion profiles may suggest novel vulnerabilities in the cancer microenvironment, which can be explored in future work.

Next, we analyzed CM from cells grown in 3D silk scaffolds. Having detected so few significant proteins in our 2D transwell system, we used additional replicates for each pBMAd donor (three pBMAd donors in total) and analyzed each donor separately. For each donor, CMs from three individual scaffolds were submitted (as technical replicates) for pBMAds alone, MM.1S alone, and co-culture conditions, and each donor was considered one biological replicate. Differences and similarities were observed between donor pBMAds (**Supplementary Figure 8A**). In our first experiment (donor 1 pBMAds, **Supplementary Tables 4–6**), 264 proteins were detected in the CMs and used to perform PCA (**Supplementary Figure 8B**). There were 35 DEPs between the groups (**Figure 7A**) in this experiment. In the second experiment using 3D scaffolds, we used two pBMAd donors (donor 2 and donor 3; **Supplementary Figure 8C**), and 108 proteins were detected in the CM samples in the groups. In donor 2 (**Supplementary Tables 7–9**), 39 DEPs were found with upregulation of PZP and IGLC3 in the co-culture compared with either cell type alone (**Figure 7B**). In donor 3 (**Supplementary Tables 10–12**), 48 DEPs were found (**Figure 7C**) including IGLC3 and AFP- two proteins that were found as significant in co-culture samples using other donors. Across all three donors, there were four proteins commonly differentially expressed: IGKC, VIM, FGA, and ITIH3.

Vimentin (VIM) was the only protein that was consistently elevated in all three pBMAd donors (versus MM.1S alone) in 3D cultures (**Figure 7**, arrows; **Supplementary Tables 4, 7, 10**), whereas the many other changed DEPs were identified but more donor-dependent. Among the three pBMAd donors, there were a total of 55 proteins significantly elevated in pBMAd cultures compared with media alone [for this analysis only, we used a more stringent fold change cut off (FC > 1.2) and p < 0.05 due to the number of proteins identified]. These proteins were enriched for members of the complement cascade and included proteins involved in glycolysis/gluconeogenesis (**Figure 8**), as well as other carbon metabolism or lipid droplet-related proteins. Having observed such differences between donors in the pBMAds alone (**Supplementary Figure 8**), we assessed and analyzed proteins changed in the 3D co-culture CM relative to the pBMAd donor media or relative to MM.1S control media (**Figures 9**). Here, we noted similarities to the pBMAds alone in terms of enriched molecules, with additional KEGG pathways highlighted in the co-culture from pBMAd donor 3 (**Figure 9**, bottom). Caution should be exercised when comparing between 2D and 3D pBMAd proteomic results, as differences could arise from different donors, media, nutrient diffusion, cell densities, cell contact, substrate stiffness, or extracellular matrix peptide signaling from silk proteins that could affect cellular adhesion or spreading. In summary, the combination of these pBMAd-MM co-culture systems (in both 2D and 3D) with proteomics assessment of CM can provide new insights into the secreted factors from pBMAds and reveal novel signaling mechanisms between these fragile cells and other cell types within the BM microenvironment.

## Discussion

In this protocol, we reveal a new method to maintain pBMAds in 3D culture and create co-cultures of pBMAds with myeloma cells to recapitulate the cancer/BM microenvironment. Our methods help address the current challenges of obtaining and culturing pBMAds, which are fragile and difficult to access and thus challenging to study *in vitro* or *ex vivo*. We integrate findings from our prior research, which generates BMAT models by differentiating MSCs from adipocytes on silk scaffolds (27) and isolating and culturing primary white adipocytes on silk scaffolds (38), to the optimal methods here in isolating pBMAds (43). This led to the new methods for culturing and imaging pBMAds and co-culturing these with other cell types. In our 3D silk scaffold pBMAd model, we were able to detect live pBMAds over time using RealTime-Glo reagent, as well as differences between donors and with different incubation times (**Supplementary Figure 2**). Future experiments should utilize this system to detect differences in pBMAds in response to tumor cell co-culture or tumor cell CM to further investigate potential effects of tumor cells on pBMAd energy production and/or cell number. These methods can be combined with luciferase- or green fluorescent protein (GFP)-expressing tumor cell lines for relative quantification of both cell types, similar to what we have described previously (27). Further optimization of different media compositions to support both cell types in culture could also be considered; here, we used both standard culture media for myeloma cells (transwell and coverslip cultures) and standard culture media for pBMAds (3D), so direct comparison between the various systems should be done with caution.

Limitations of this study include donor-to-donor variability in samples, challenges in availability/number donors (healthy, non-myeloma donors and myeloma patient donors) and difficulty in isolating RNA from pBMAds, as we experienced unusually low RNA yields using standard techniques. While we were able to isolate a small amount of total RNA from these samples and amplify higher abundance targets such as housekeeping genes [*ACTB* and *RPLP0* (data not shown)] and highly expressed adipocyte markers (*FABP4*), we were only able to amplify other desirable targets in two of the four donor samples examined. Utilization of alternative methods to avoid saturating RNA binding columns with lipid, and avoidance of trizol-based extraction reagents (35, 44, 45) would likely improve yield in future studies. Further development of these methods is required to improve the utility of working with these cells in culture and to advance the field. However, the utilization of proteomics-based analyses allowed us to steer away from these limitations and further investigate the relationship between myeloma cells and BMAds. T-test comparisons for proteomics data were executed using either GraphPad Prism or MarkerView software, which is specifically designed for the handling of mass spectrometry data; however, future studies could implement more stringent statistical comparisons including false discovery rate correction.

Although we did not utilize these methods, ImageJ or other software platforms can be used to quantify adipocytes and measure lipid droplet size in both 2D and 3D cultures. To characterize the effects of pBMAd co-culture on MM cells in 3D culture, magnetically activated cell sorting can be implemented to gently collect CD138^+^ cells after rinsing tumor cells off the scaffolds, for downstream analyses including flow-based apoptosis, proliferation, and cell cycle assays. Alternatively, more targeted approaches, such as enzyme-linked immunosorbent assays (ELISAs), could be used to specifically examine the effects on soluble adipokines and signaling molecules present in CM.

Proteomic analysis of CM collected from single-cell types (pBMAds or MM cell lines) or cells in co-culture revealed a wealth of new information about secretory factors and potential signaling molecules produced by these cells. In 2D transwell co-cultures, we detected elevated levels of AFP and fibromodulin (FMOD). Elevated AFP levels have been associated with metabolic syndrome (46), and stromal cells differentiating into adipocytes (47, 48) suggesting that mature BMAds may also produce this protein and it may be upregulated upon exposure to myeloma cells. The extracellular matrix protein FMOD, which is also increased in transwell co-cultures relative to the pBMAds alone, regulates BMSC differentiation through regulation of the BMP2 pathway, modulating chromatin structure to provide access for the RUNX2/PPARG transcription factors to bind and regulate BMSC lineage commitment (49). Critically, FMOD is not expressed in myeloma cells (50), suggesting that this protein does indeed come from the pBMAd cultures. Together, these two data points suggest either that there is stromal contamination in the co-culture system (and that these stromata are responding to the myeloma cells) or that myeloma cells might stimulate de-differentiation of adipocytes in culture. Future studies should interrogate this.

In 3D culture, we were able to detect levels of the intermediate-sized filament VIM in the culture media from all 3 pBMAd donors. Interestingly, lipid droplets in adipocytes are often biochemically associated with VIM, with VIM attaching to perlipins inside the cell (51, 52) and may be involved in lipid trafficking (51). Studies have linked VIM to exosomes derived from adipocyte progenitors (53, 54), which could account for the presence of this protein in the CM from pBMAds. Indeed, recent studies suggest that adipocyte-derived exosomes may protect MM cells from chemotherapy-induced apoptosis (55), and although we did not directly look for exosomes in the media, we did find a number of cytoskeletal-associated proteins in the pBMAds alone and the co-culture media, which could be consistent with the presence of exosomes.

In donor 1, three proteins were significantly upregulated in the co-culture condition compared with either cell type alone including LDHB, YWHAZ, and EEF1A2. LDHB is upregulated in cancer associated adipocytes in the breast cancer microenvironment, suggesting that cancer cells, including myeloma cells, could modulate lactate metabolism in the microenvironment (56)—a process that might be critical in patient responses to carfilzomib (57). In the 3D co-culture using cells from donor 2, PZP and IGLC3 were significantly upregulated compared with both cell types alone. Although PZP may play a role in energy conversion in adipocytes (58), very little is known about its role in either cell type tested in our experiments. Interestingly, LAC3 (Iglc3) was upregulated in extracellular vesicles derived from the BM of myeloma-bearing mice, compared with control extracellular vesicles (59), and is involved in complement activation (59). In the CM from the donor 3 co-culture scaffolds, we observed significant differences in 14 proteins compared with both cell types alone, including IGLC3 and AFP—two proteins that were also significant in co-culture samples using other donors.

Our data from pBMAds add to the burgeoning datasets, suggesting that these cells, once thought of as static, space-filling cells, may be critical regulators of bone, angiogenesis, and immune function. Our preliminary investigation comparing transwell co-cultures and 3D silk scaffold cultures suggests that pBMAds likely secrete different factors based on their culture method; future directions should continue to optimize culture conditions of these fragile cells for use *in vivo*. In addition, future studies should include a side-by-side comparison of BMSC-derived BMAds with pBMAds to better understand any potential differences between the two *in vitro*. Utilization of larger primary samples (perhaps from amputations, cadavers, etc.) would also be beneficial in future studies to increase the signal strength in RNA and proteomic analyses.

We found that pBMAds can be grown in both ceiling cultures (using the undersides of coverslips and of transwells as the ceiling substrates for adhesion) and in 3D silk scaffolds. Both culture systems can be used to detect secreted molecules, investigate tumor cell-adipocyte interactions, and visualize phenotypic changes in cells. These models will help researchers to investigate tumor-adipocyte microenvironment and bidirectional signaling between the cells, which may lead to the development of new therapies or avenues for intervention in cancer progression. In summary, we provide methods to ease the use of pBMAds rather than BMSC-derived adipocytes that can lead to more translationally relevant findings to efficiently investigate how adipocytes support tumor cells and how tumor cells hijack BMAds for their own purposes.

## Data availability statement

The mass spectrometry proteomics data have been deposited to the ProteomeXchange Consortium via the PRIDE partner repository with the dataset identifier PXD032753 (60). http://www.ebi.ac.uk/pride.

## Author contributions

Conception and design (HF, RC, and MR). Development of methodology (HF, RC, MF, and MR). Acquisition of data (HF, RC, MF, RD, CG, CV, and MR). Analysis and interpretation of data (HF, RDI, CV, and MR). Writing, review, and/or revision of the manuscript (HF and MR wrote the manuscript, all authors contributed to review and revision). Administrative (HF and MR), technical (HF, MF, CV, CG, and MR), or material support (HF and MF) and study supervision (HF and MR). All authors contributed to the article and approved the submitted version.

## References

[1] Giannakoulas N Ntanasis-Stathopoulos I Terpos E . The role of marrow microenvironment in the growth and development of malignant plasma cells in multiple myeloma. Int J Mol Sci (2021) 22:4462. doi: 10.3390/IJMS22094462 33923357 PMC8123209

[2] Fairfield H Falank C Avery L Reagan MR . Multiple myeloma in the marrow: pathogenesis and treatments. Ann N Y Acad Sci (2016) 1364:32–51. doi: 10.1111/nyas.13038 27002787 PMC4806534

[3] Reagan MR Liaw L Rosen CJ Ghobrial IM . Dynamic interplay between bone and multiple myeloma: Emerging roles of the osteoblast. Bone (2015) 75:161–9. doi: 10.1016/j.bone.2015.02.021 PMC4580250 25725265

[4] Delgado-Calle J Anderson J Cregor MD Condon KW Kuhstoss SA Plotkin LI . Genetic deletion of sost or pharmacological inhibition of sclerostin prevent multiple myeloma-induced bone disease without affecting tumor growth. Leukemia (2017) 31:2686–94. doi: 10.1038/leu.2017.152 PMC5699973 28529307

[5] McDonald MM Reagan MR Youlten SE Mohanty ST Seckinger A Terry RL . Inhibiting the osteocyte-specific protein sclerostin increases bone mass and fracture resistance in multiple myeloma. Blood (2017) 129:3452–64. doi: 10.1182/blood-2017-03-773341 PMC5492093 28515094

[6] Ho M Chen T Liu J Dowling P Hideshima T Zhang L . Targeting histone deacetylase 3 (HDAC3) in the bone marrow microenvironment inhibits multiple myeloma proliferation by modulating exosomes and IL-6 trans-signaling. Leukemia (2020) 34:196–209. doi: 10.1038/s41375-019-0493-x PMC6883144 31142847

[7] Delgado-Calle J Anderson J Cregor MD Hiasa M Chirgwin JM Carlesso N . Bidirectional notch signaling and osteocyte-derived factors in the bone marrow microenvironment promote tumor cell proliferation and bone destruction in multiple myeloma. Cancer Res (2016) 76:1089–100. doi: 10.1158/0008-5472.CAN-15-1703 PMC4775415 26833121

[8] Roccaro AMM Mishima Y Sacco A Moschetta M Tai Y-T Shi J . CXCR4 regulates extra-medullary myeloma through epithelial-mesenchymal-transition-like transcriptional activation. Cell Rep (2015) 12:622–35. doi: 10.1016/j.celrep.2015.06.059 PMC4961259 26190113

[9] Trotter TN Gibson JT Sherpa TL Gowda PS Peker D Yang Y . Adipocyte-lineage cells support growth and dissemination of multiple myeloma in bone. Am J Pathol (2016) 186:3054–63. doi: 10.1016/j.ajpath.2016.07.012 PMC5222958 27648615

[10] Caers J Deleu S Belaid Z De Raeve H Van Valckenborgh E De Bruyne E . Neighboring adipocytes participate in the bone marrow microenvironment of multiple myeloma cells. Leukemia (2007) 21:1580–4. doi: 10.1038/sj.leu.2404658 17377589

[11] Liu Z Xu J He J Liu H Lin P Wan X . Mature adipocytes in bone marrow protect myeloma cells against chemotherapy through autophagy activation. Oncotarget (2015) 6:34329–41. doi: 10.18632/oncotarget.6020 PMC4741456 26455377

[12] Cawthorn WP Scheller EL Learman BS Parlee SD Simon BR Mori H . Bone marrow adipose tissue is an endocrine organ that contributes to increased circulating adiponectin during caloric restriction. Cell Metab (2014) 20:368–75. doi: 10.1016/j.cmet.2014.06.003 PMC4126847 24998914

[13] Horowitz MC Berry R Holtrup B Sebo Z Nelson T Fretz JA . Bone marrow adipocytes. Adipocyte (2017) 6:193–204. doi: 10.1080/21623945.2017.1367881 28872979 PMC5638373

[14] Fan Y Hanai J Le PT Bi R Maridas D DeMambro V . Parathyroid hormone directs bone marrow mesenchymal cell fate. Cell Metab (2017) 25:661–72. doi: 10.1016/j.cmet.2017.01.001 PMC5342925 28162969

[15] Zhou BO Yu H Yue R Zhao Z Rios JJ Naveiras O . Bone marrow adipocytes promote the regeneration of stem cells and haematopoiesis by secreting SCF. Nat Cell Biol (2017) 19:891–903. doi: 10.1038/ncb3570 28714970 PMC5536858

[16] Boyd AL Reid JC Salci KR Aslostovar L Benoit YD Shapovalova Z . Acute myeloid leukaemia disrupts endogenous myelo-erythropoiesis by compromising the adipocyte bone marrow niche. Nat Cell Biol (2017) 19:1336–47. doi: 10.1038/ncb3625 29035359

[17] Dirat B Bochet L Dabek M Daviaud D Dauvillier S Majed B . Cancer-associated adipocytes exhibit an activated phenotype and contribute to breast cancer invasion. Cancer Res (2011) 71:2455–65. doi: 10.1158/0008-5472.CAN-10-3323 21459803

[18] Benova A Tencerova M . Obesity-induced changes in bone marrow homeostasis. Front Endocrinol (Lausanne) (2020) 11:294. doi: 10.3389/fendo.2020.00294 32477271 PMC7235195

[19] Aaron N Costa S Rosen CJ Qiang L . The implications of bone marrow adipose tissue on inflammaging. Front Endocrinol (Lausanne) (2022) 0:853765. doi: 10.3389/FENDO.2022.853765 PMC8962663 35360075

[20] Marinac CR Suppan CA Giovannucci E Song M Kvaerner AS Townsend MK . Elucidating under-studied aspects of the link between obesity and multiple myeloma: Weight pattern, body shape trajectory, and body fat distribution. J Natl Cancer Inst (2019) 3. doi: 10.1093/jncics/pkz044 PMC6699596 31448358

[21] Teras LR Kitahara CM Birmann BM Hartge PA Wang SS Robien K . Body size and multiple myeloma mortality: a pooled analysis of 20 prospective studies. Br J Haematol (2014) 166:667–76. doi: 10.1111/bjh.12935 PMC4134758 24861847

[22] Corre J Munshi NC Avet-Loiseau H . Risk factors in multiple myeloma: is it time for a revision? Blood (2021) 137:16–9. doi: 10.1182/BLOOD.2019004309 PMC7808011 33024991

[23] Bullwinkle EM Parker MD Bonan NF Falkenberg LG Davison SP DeCicco-Skinner KL . Adipocytes contribute to the growth and progression of multiple myeloma: Unraveling obesity related differences in adipocyte signaling. Cancer Lett (2016) 380:114–21. doi: 10.1016/j.canlet.2016.06.010 27317873

[24] Liu H He J Koh SP Zhong Y Liu Z Wang Z . Reprogrammed marrow adipocytes contribute to myeloma-induced bone disease. Sci Transl Med (2019) 11:eaau9087. doi: 10.1126/scitranslmed.aau9087 31142679 PMC6999853

[25] Morris EV Suchacki KJ Hocking J Cartwright R Sowman A Gamez B . Myeloma cells down-regulate adiponectin in bone marrow adipocytes *Via* TNF-alpha. J Bone Miner Res (2019) 35:942–55. doi: 10.1002/jbmr.3951 PMC9328417 31886918

[26] Fairfield H Dudakovic A Khatib CM Farrell M Costa S Falank C . Myeloma-modified adipocytes exhibit metabolic dysfunction and a senescence-associated secretory phenotype. Cancer Res (2021) 81:634–47. doi: 10.1158/0008-5472.CAN-20-1088 PMC7854508 33218968

[27] Fairfield H Falank C Farrell M Vary C Boucher JMJM Driscoll H . Development of a 3D bone marrow adipose tissue model. Bone (2018) 118:77–88. doi: 10.1016/j.bone.2018.01.023 29366838 PMC6062483

[28] Fairfield H Costa S Falank C Farrell M Murphy C D’Amico A . Multiple myeloma cells alter adipogenesis, increase senescence-related and inflammatory gene transcript expression, and alter metabolism in preadipocytes. Front Oncol (2021) 10:584683. doi: 10.3389/fonc.2020.584683 33680918 PMC7930573

[29] Chan CKF Seo EY Chen JY Lo D McArdle A Sinha R . Identification and specification of the mouse skeletal stem cell. Cell (2015) 160:285–98. doi: 10.1016/j.cell.2014.12.002 PMC4297645 25594184

[30] Owen M Friedenstein AJ . Stromal stem cells: marrow-derived osteogenic precursors. Ciba Found Symp (1988) 136:42–60. doi: 10.1002/9780470513637.CH4 3068016

[31] Suchacki KJ Tavares AAS Mattiucci D Scheller EL Papanastasiou G Gray C . Bone marrow adipose tissue is a unique adipose subtype with distinct roles in glucose homeostasis. Nat Commun (2020) 11. doi: 10.1038/s41467-020-16878-2 PMC7303125 32555194

[32] Attané C Estève D Chaoui K Iacovoni JS Corre J Moutahir M . Human bone marrow is comprised of adipocytes with specific lipid metabolism. Cell Rep (2020) 30:949–958.e6. doi: 10.1016/J.CELREP.2019.12.089 31995765

[33] Poloni A Maurizi G Serrani F Mancini S Zingaretti MC Frontini A . Molecular and functional characterization of human bone marrow adipocytes. Exp Hematol (2013) 41:558–566.e2. doi: 10.1016/j.exphem.2013.02.005 23435314

[34] Goto H Hozumi A Osaki M Fukushima T Sakamoto K Yonekura A . Primary human bone marrow adipocytes support TNF-α-induced osteoclast differentiation and function through RANKL expression. Cytokine (2011) 56:662–8. doi: 10.1016/J.CYTO.2011.09.005 21963155

[35] Miggitsch C Meryk A Naismith E Pangrazzi L Ejaz A Jenewein B . Human bone marrow adipocytes display distinct immune regulatory properties. EBioMedicine (2019) 46:387–98. doi: 10.1016/j.ebiom.2019.07.023 PMC6711052 31327694

[36] Ferland-McCollough D Maselli D Spinetti G Sambataro M Sullivan N Blom A . MCP-1 feedback loop between adipocytes and mesenchymal stromal cells causes fat accumulation and contributes to hematopoietic stem cell rarefaction in the bone marrow of patients with diabetes. Diabetes (2018) 67:1380–94. doi: 10.2337/DB18-0044 29703845

[37] Li Z Liu H He J Wang Z Yin Z You G . Acetyl-CoA synthetase 2: A critical linkage in obesity-induced tumorigenesis in myeloma. Cell Metab (2021) 33:78–93.e7. doi: 10.1016/j.cmet.2020.12.011 33406405 PMC7799390

[38] Abbott RD Wang RY Reagan MR Chen Y Borowsky FE Zieba A . The use of silk as a scaffold for mature, sustainable unilocular adipose 3D tissue engineered systems. Adv Healthc Mater (2016) 5:1667–77. doi: 10.1002/adhm.201600211 PMC4982640 27197588

[39] Murphy CS Liaw L Reagan MR . *In vitro* tissue-engineered adipose constructs for modeling disease. BMC BioMed Eng (2019) 1:27. doi: 10.1186/s42490-019-0027-7 32133436 PMC7055683

[40] Dadwal U Falank C Fairfield H Linehan S Rosen CJ Kaplan DL . Tissue-engineered 3D cancer-in-bone modeling: silk and PUR protocols. Bonekey Rep (2016) 5:842. doi: 10.1038/bonekey.2016.75 27790370 PMC5070496

[41] Chaudhuri O Koshy ST Branco Da Cunha C Shin JW Verbeke CS Allison KH . Extracellular matrix stiffness and composition jointly regulate the induction of malignant phenotypes in mammary epithelium. Nat Mater (2014) 13:970–8. doi: 10.1038/nmat4009 24930031

[42] Schilling B Gibson BW Hunter CL . Generation of high-quality SWATH® acquisition data for label-free quantitative proteomics studies using tripleTOF® mass spectrometers. Methods Mol Biol (2017) 1550:223–33. doi: 10.1007/978-1-4939-6747-6_16/COVER PMC5669615 28188533

[43] Lucas S Tencerova M von der Weid B Andersen TL Attané C Behler-Janbeck F . Guidelines for biobanking of bone marrow adipose tissue and related cell types: Report of the biobanking working group of the international bone marrow adiposity society. Front Endocrinol (Lausanne) (2021) 12:744527. doi: 10.3389/fendo.2021.744527 34646237 PMC8503265

[44] Hemmrich K Denecke B Paul NE Hoffmeister D Pallua N . RNA Isolation from adipose tissue: An optimized procedure for high RNA yield and integrity. Lab Med (2010) 41:104–6. doi: 10.1309/LMFSBPUOA19MH5BV

[45] Nouvel A Laget J Duranton F Leroy J Desmetz C Servais MD . Optimization of RNA extraction methods from human metabolic tissue samples of the COMET biobank. Sci Rep (2021) 11:1–12. doi: 10.1038/s41598-021-00355-x 34697345 PMC8545963

[46] Chen Y Zhao Y Feng L Zhang J Zhang J Feng G . Association between alpha-fetoprotein and metabolic syndrome in a Chinese asymptomatic population: A cross-sectional study. Lipids Health Dis (2016) 15:1–9. doi: 10.1186/S12944-016-0256-X/FIGURES/4 27121855 PMC4848775

[47] Seo MJ Suh SY Bae YC Jung JS . Differentiation of human adipose stromal cells into hepatic lineage *in vitro* and *in vivo* . Biochem Biophys Res Commun (2005) 328:258–64. doi: 10.1016/J.BBRC.2004.12.158 15670778

[48] Obara C Tomiyama KI Takizawa K Islam R Yasuda T Gotoh T . Characteristics of three-dimensional prospectively isolated mouse bone marrow mesenchymal stem/stromal cell aggregates on nanoculture plates. Cell Tissue Res (2016) 366:113–27. doi: 10.1007/S00441-016-2405-Y 27100525

[49] Li CJ Xiao Y Yang M Su T Sun X Guo Q . Long noncoding RNA bmncr regulates mesenchymal stem cell fate during skeletal aging. J Clin Invest (2018) 128:5219–21. doi: 10.1172/JCI99044 PMC6264619 30352426

[50] Mikaelsson E Danesh-Manesh AH Lüppert A Jeddi-Tehrani M Rezvany MR Sharifian RA . Fibromodulin, an extracellular matrix protein: characterization of its unique gene and protein expression in b-cell chronic lymphocytic leukemia and mantle cell lymphoma. Blood (2005) 105:4828–35. doi: 10.1182/BLOOD-2004-10-3941 15741214

[51] Schweitzer SC Evans RM . Vimentin and lipid metabolism. Subcell Biochem (1998) 31:437–62. 9932502

[52] Heid H Rickelt S Zimbelmann R Winter S Schumacher H Dörflinger Y . On the formation of lipid droplets in human adipocytes: the organization of the perilipin-vimentin cortex. PloS One (2014) 9(2):e90386. doi: 10.1371/JOURNAL.PONE.0090386 PMC3938729 24587346

[53] Parvanian S Yan F Su D Coelho-Rato LS Venu AP Yang P . Exosomal vimentin from adipocyte progenitors accelerates wound healing. Cytoskeleton (Hoboken) (2020) 77:399–413. doi: 10.1002/CM.21634 32978896

[54] Parvanian S Zha H Su D Xi L Jiu Y Chen H . Exosomal vimentin from adipocyte progenitors protects fibroblasts against osmotic stress and inhibits apoptosis to enhance wound healing. Int J Mol Sci (2021) 22:4678. doi: 10.3390/ijms22094678 PMC8125065 33925176

[55] Wang Z He J Bach DH Huang YH Li Z Liu H . Induction of m6A methylation in adipocyte exosomal LncRNAs mediates myeloma drug resistance. J Exp Clin Cancer Res (2022) 41:1–18. doi: 10.1186/S13046-021-02209-W/FIGURES/8 34980213 PMC8722039

[56] Kalezic A Udicki M Galic BS Aleksic M Korac A Jankovic A . Lactate metabolism in breast cancer microenvironment: Contribution focused on associated adipose tissue and obesity. Int J Mol Sci (2020) 21:1–13. doi: 10.3390/IJMS21249676 PMC7766866 33353120

[57] Tantawy M Chekka LM Huang Y Garrett TJ Singh S Shah CP . Lactate dehydrogenase b and pyruvate oxidation pathway associated with carfilzomib-related cardiotoxicity in multiple myeloma patients: Result of a multi-omics integrative analysis. Front Cardiovasc Med (2021) 8:645122. doi: 10.3389/FCVM.2021.645122 33996940 PMC8116486

[58] Jiang X Lin J Dong M Liu X Huang Y Zhang H . Overexpression of pregnancy zone protein in fat antagonizes diet-induced obesity under an intermittent fasting regime. Front Physiol (2022) 13:950619/BIBTEX. doi: 10.3389/FPHYS.2022.950619/BIBTEX 36051914 PMC9424687

[59] Lopes R Caetano J Barahona F Pestana C Ferreira BV Lourenço D . Multiple myeloma-derived extracellular vesicles modulate the bone marrow immune microenvironment. Front Immunol (2022) 13:909880. doi: 10.3389/FIMMU.2022.909880 35874665 PMC9302002

[60] Perez-Riverol Y Bai J Bandla C García-Seisdedos D Hewapathirana S Kamatchinathan S . The PRIDE database resources in 2022: A hub for mass spectrometry-based proteomics evidences. Nucleic Acids Res (2022) 50:D543–52. doi: 10.1093/NAR/GKAB1038 PMC8728295 34723319

