# Supplementary material for: Development and characterization of three cell culture systems to investigate the relationship between primary bone marrow adipocytes and myeloma cells
Source: Front Oncol. 2023 Jan 11;12:912834. doi: 10.3389/fonc.2022.912834 (PMC9874147; doi:10.3389/fonc.2022.912834)
Supplement: Supplementary file 1 [file Presentation_1.pptx]

## Slide 1
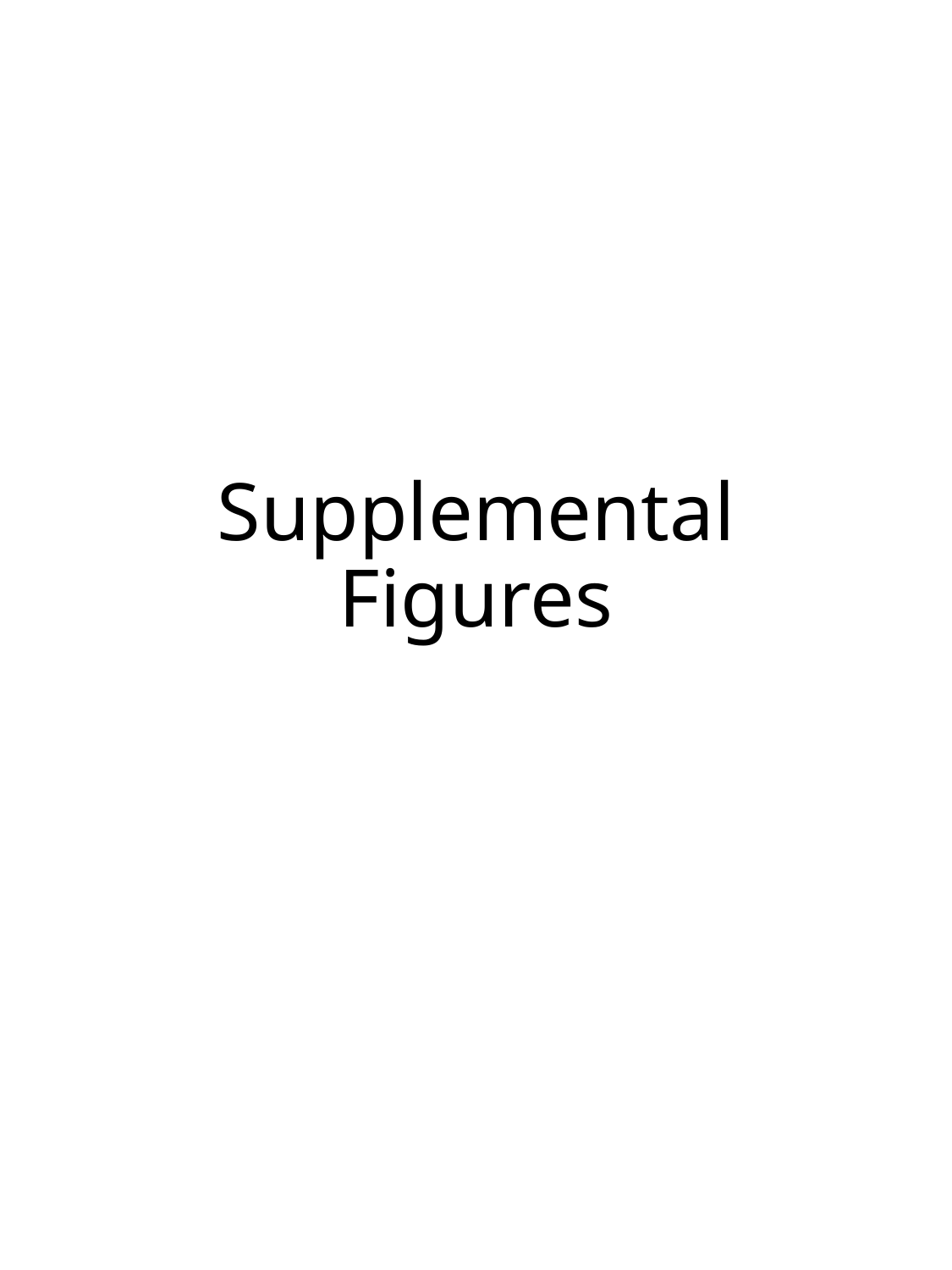

# Supplemental Figures

## Slide 2
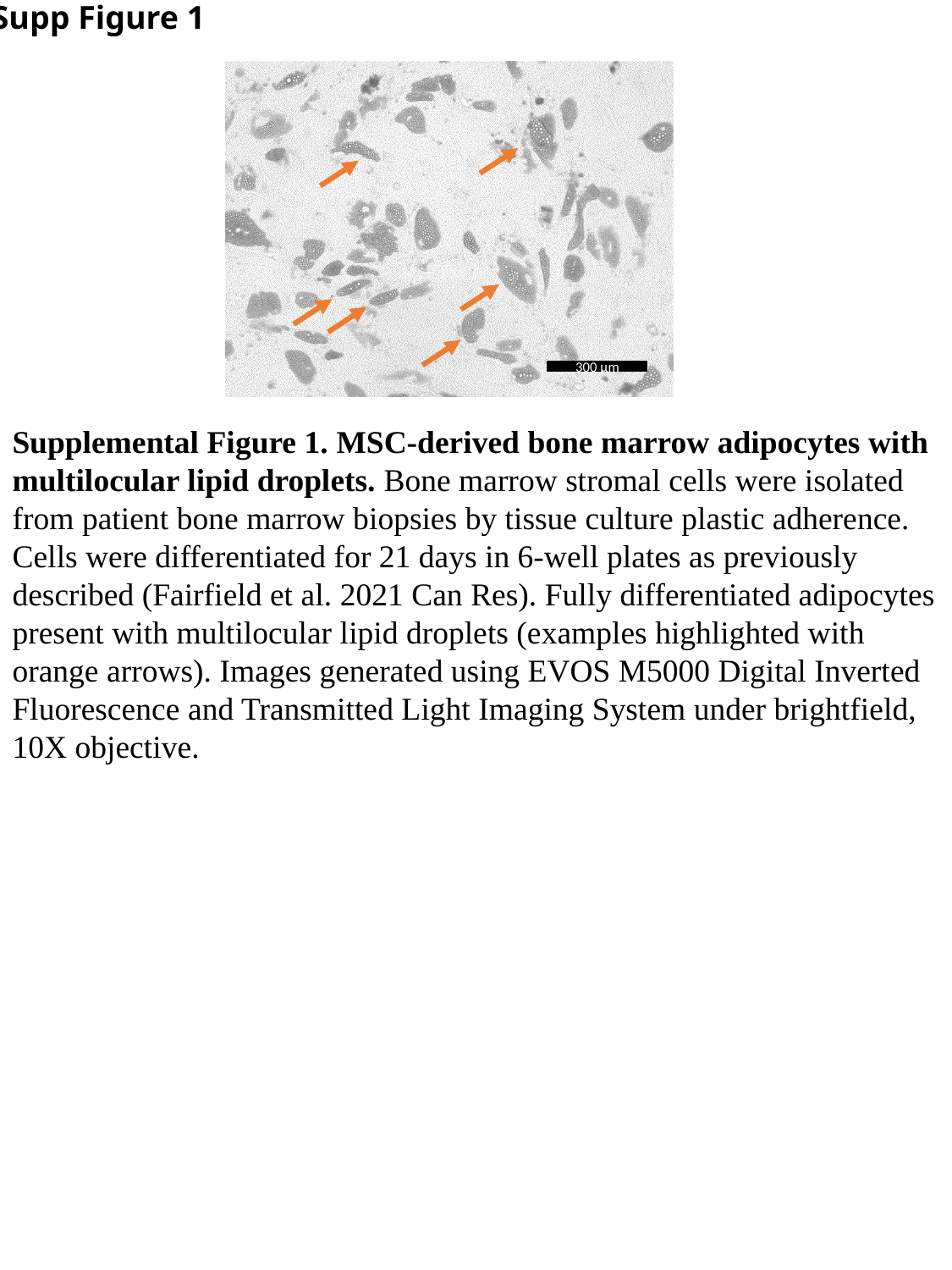

Supp Figure 1
300 µm
Supplemental Figure 1. MSC-derived bone marrow adipocytes with multilocular lipid droplets. Bone marrow stromal cells were isolated from patient bone marrow biopsies by tissue culture plastic adherence. Cells were differentiated for 21 days in 6-well plates as previously described (Fairfield et al. 2021 Can Res). Fully differentiated adipocytes present with multilocular lipid droplets (examples highlighted with orange arrows). Images generated using EVOS M5000 Digital Inverted Fluorescence and Transmitted Light Imaging System under brightfield, 10X objective.

## Slide 3
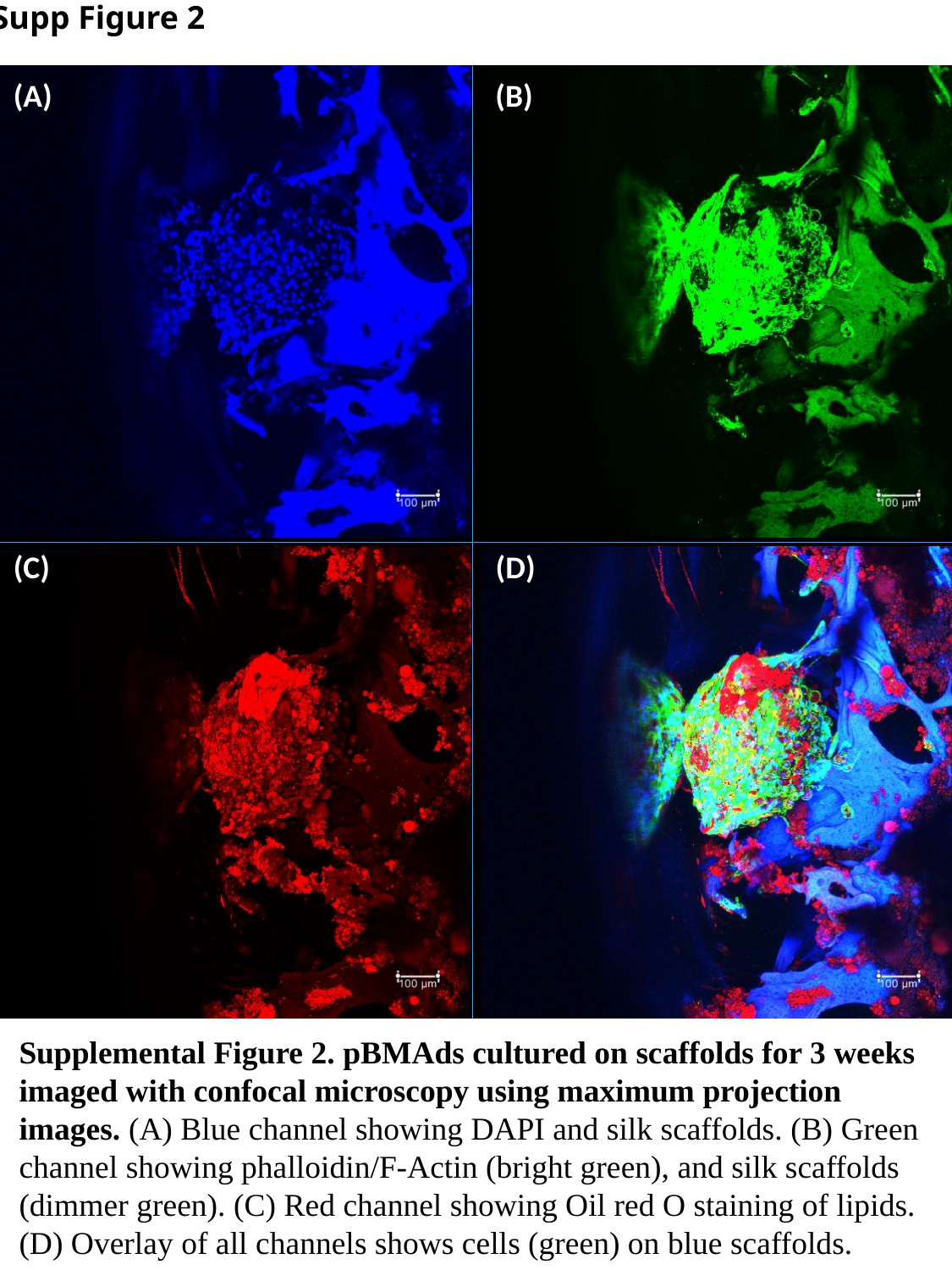

Supp Figure 2
(A)
(B)
(C)
(D)
Supplemental Figure 2. pBMAds cultured on scaffolds for 3 weeks imaged with confocal microscopy using maximum projection images. (A) Blue channel showing DAPI and silk scaffolds. (B) Green channel showing phalloidin/F-Actin (bright green), and silk scaffolds (dimmer green). (C) Red channel showing Oil red O staining of lipids. (D) Overlay of all channels shows cells (green) on blue scaffolds.

## Slide 4
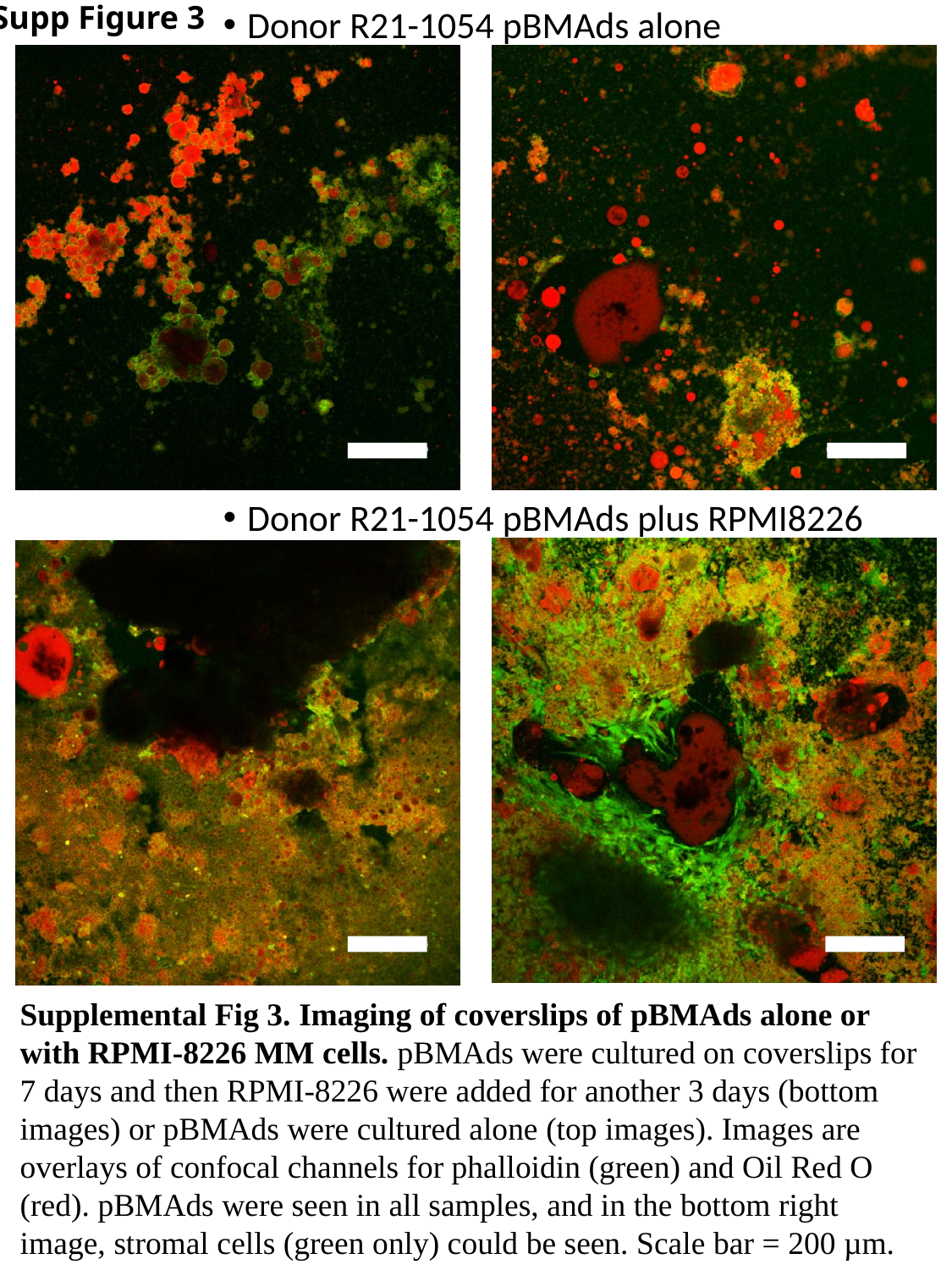

Donor R21-1054 pBMAds alone
Supp Figure 3
Donor R21-1054 pBMAds plus RPMI8226
Supplemental Fig 3. Imaging of coverslips of pBMAds alone or with RPMI-8226 MM cells. pBMAds were cultured on coverslips for 7 days and then RPMI-8226 were added for another 3 days (bottom images) or pBMAds were cultured alone (top images). Images are overlays of confocal channels for phalloidin (green) and Oil Red O (red). pBMAds were seen in all samples, and in the bottom right image, stromal cells (green only) could be seen. Scale bar = 200 µm.

## Slide 5
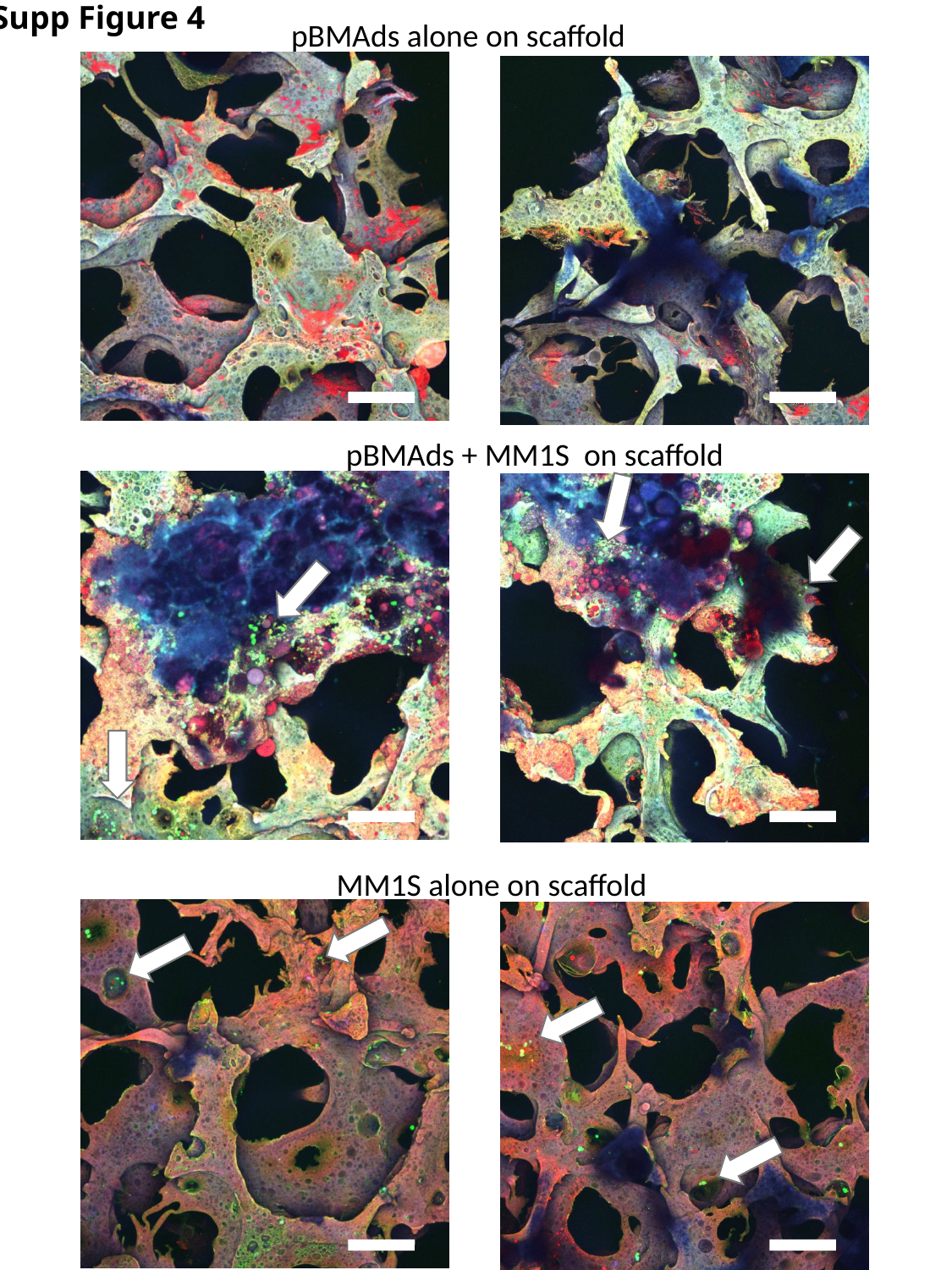

Supp Figure 4
pBMAds alone on scaffold
pBMAds + MM1S on scaffold
MM1S alone on scaffold

## Slide 6
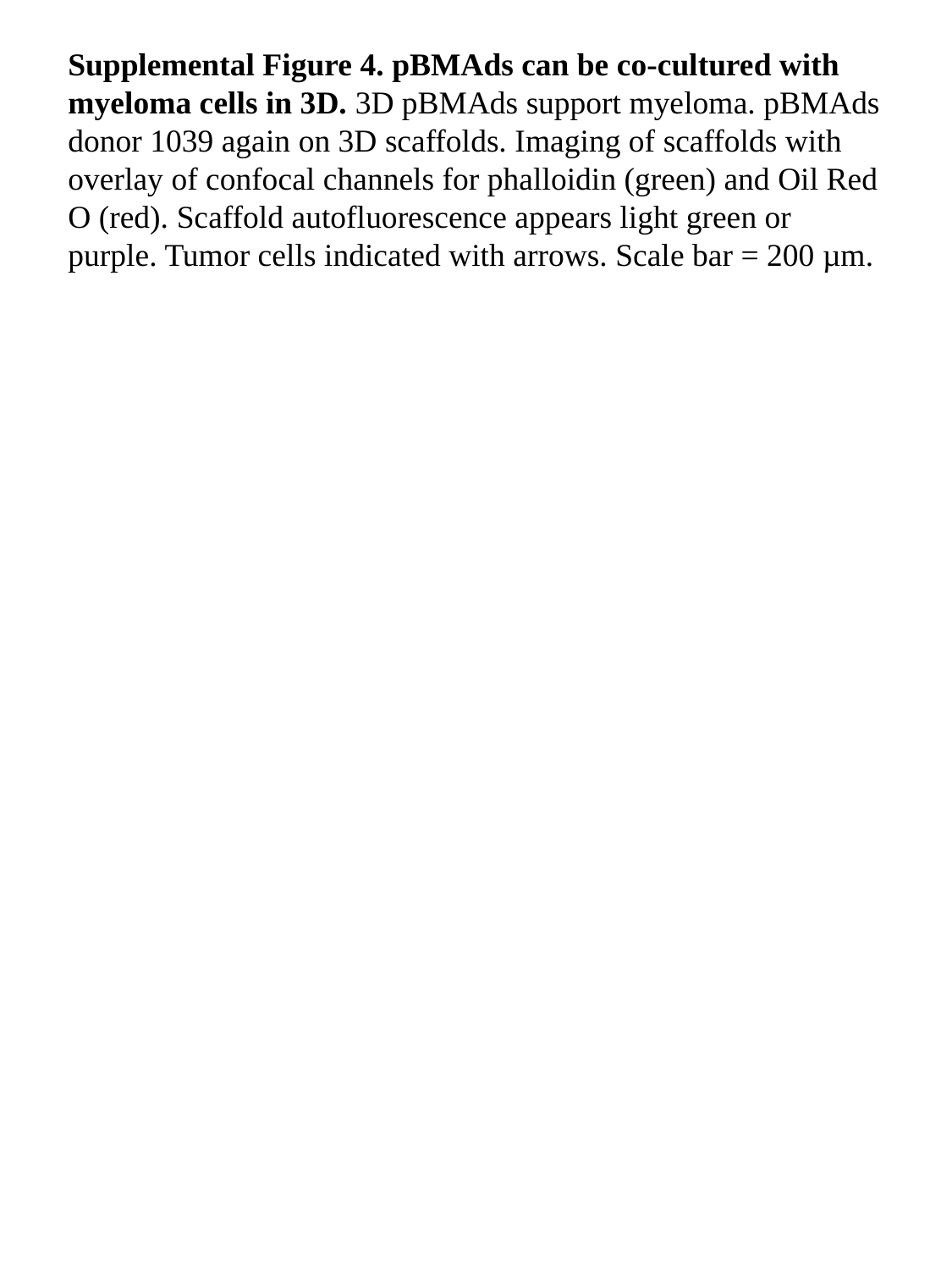

Supplemental Figure 4. pBMAds can be co-cultured with myeloma cells in 3D. 3D pBMAds support myeloma. pBMAds donor 1039 again on 3D scaffolds. Imaging of scaffolds with overlay of confocal channels for phalloidin (green) and Oil Red O (red). Scaffold autofluorescence appears light green or purple. Tumor cells indicated with arrows. Scale bar = 200 µm.

## Slide 7
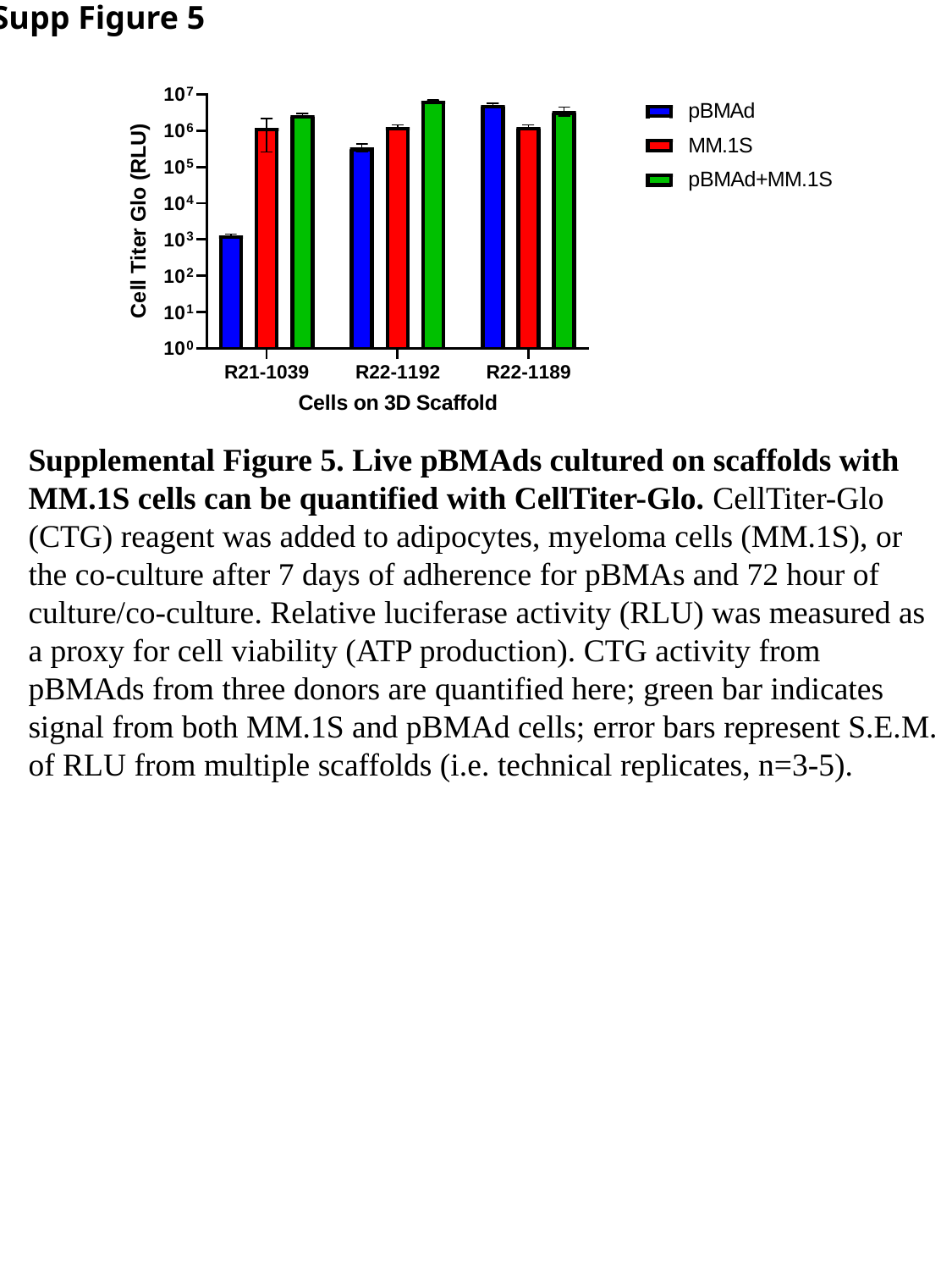

Supp Figure 5
Supplemental Figure 5. Live pBMAds cultured on scaffolds with MM.1S cells can be quantified with CellTiter-Glo. CellTiter-Glo (CTG) reagent was added to adipocytes, myeloma cells (MM.1S), or the co-culture after 7 days of adherence for pBMAs and 72 hour of culture/co-culture. Relative luciferase activity (RLU) was measured as a proxy for cell viability (ATP production). CTG activity from pBMAds from three donors are quantified here; green bar indicates signal from both MM.1S and pBMAd cells; error bars represent S.E.M. of RLU from multiple scaffolds (i.e. technical replicates, n=3-5).

## Slide 8
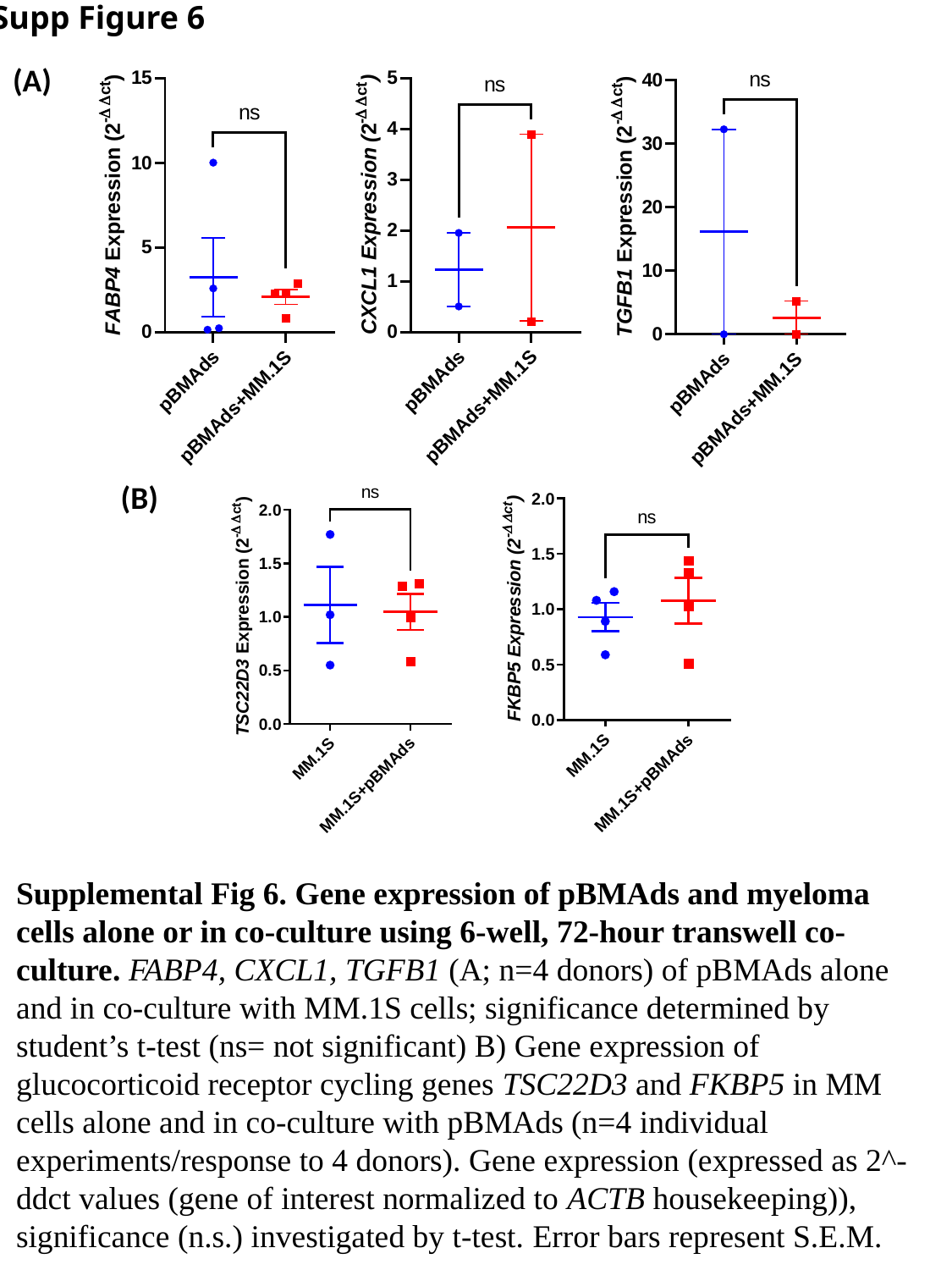

Supp Figure 6
(A)
(B)
Supplemental Fig 6. Gene expression of pBMAds and myeloma cells alone or in co-culture using 6-well, 72-hour transwell co-culture. FABP4, CXCL1, TGFB1 (A; n=4 donors) of pBMAds alone and in co-culture with MM.1S cells; significance determined by student’s t-test (ns= not significant) B) Gene expression of glucocorticoid receptor cycling genes TSC22D3 and FKBP5 in MM cells alone and in co-culture with pBMAds (n=4 individual experiments/response to 4 donors). Gene expression (expressed as 2^-ddct values (gene of interest normalized to ACTB housekeeping)), significance (n.s.) investigated by t-test. Error bars represent S.E.M.

## Slide 9
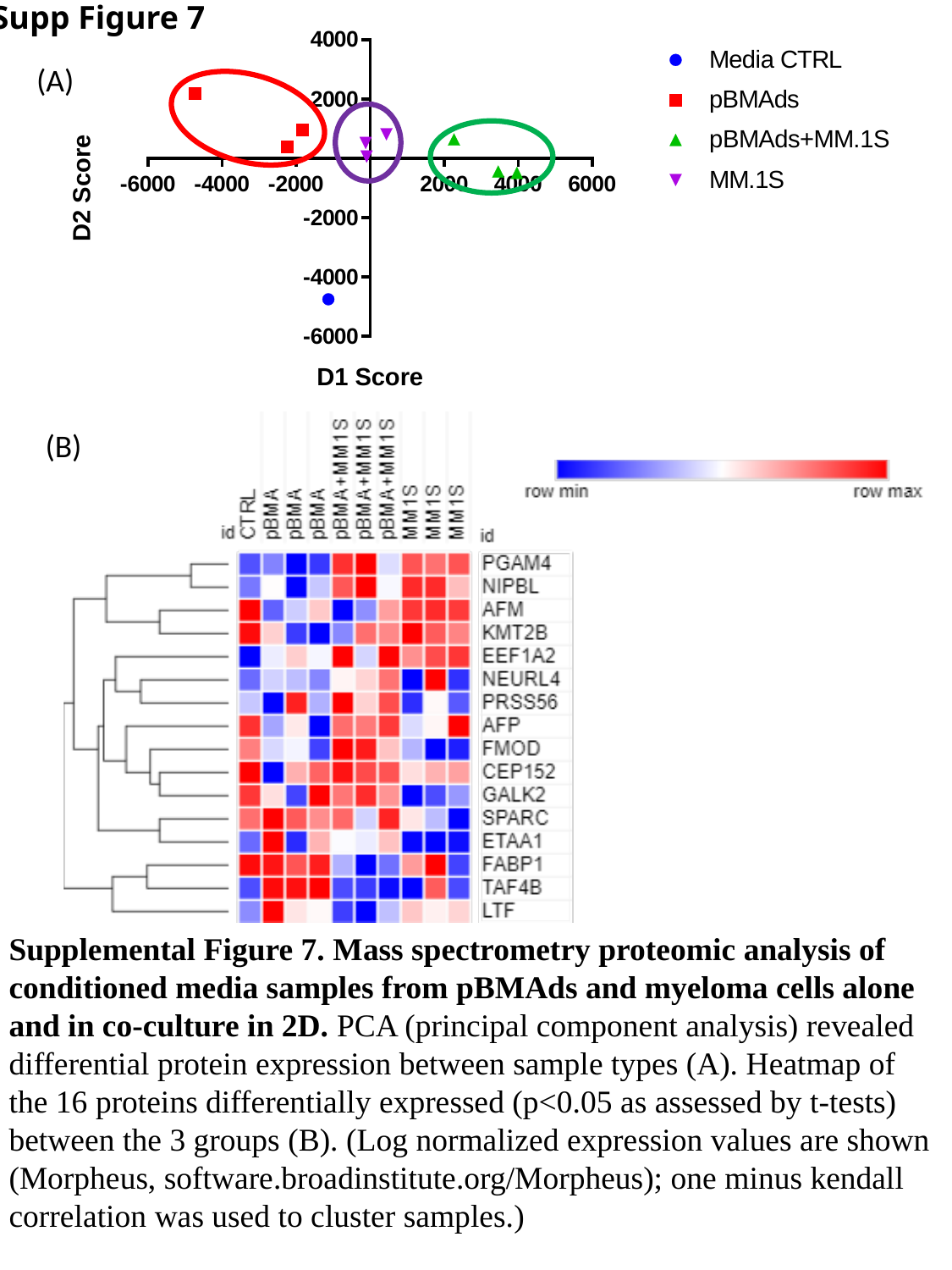

Supp Figure 7
(A)
(B)
Supplemental Figure 7. Mass spectrometry proteomic analysis of conditioned media samples from pBMAds and myeloma cells alone and in co-culture in 2D. PCA (principal component analysis) revealed differential protein expression between sample types (A). Heatmap of the 16 proteins differentially expressed (p<0.05 as assessed by t-tests) between the 3 groups (B). (Log normalized expression values are shown (Morpheus, software.broadinstitute.org/Morpheus); one minus kendall correlation was used to cluster samples.)

## Slide 10
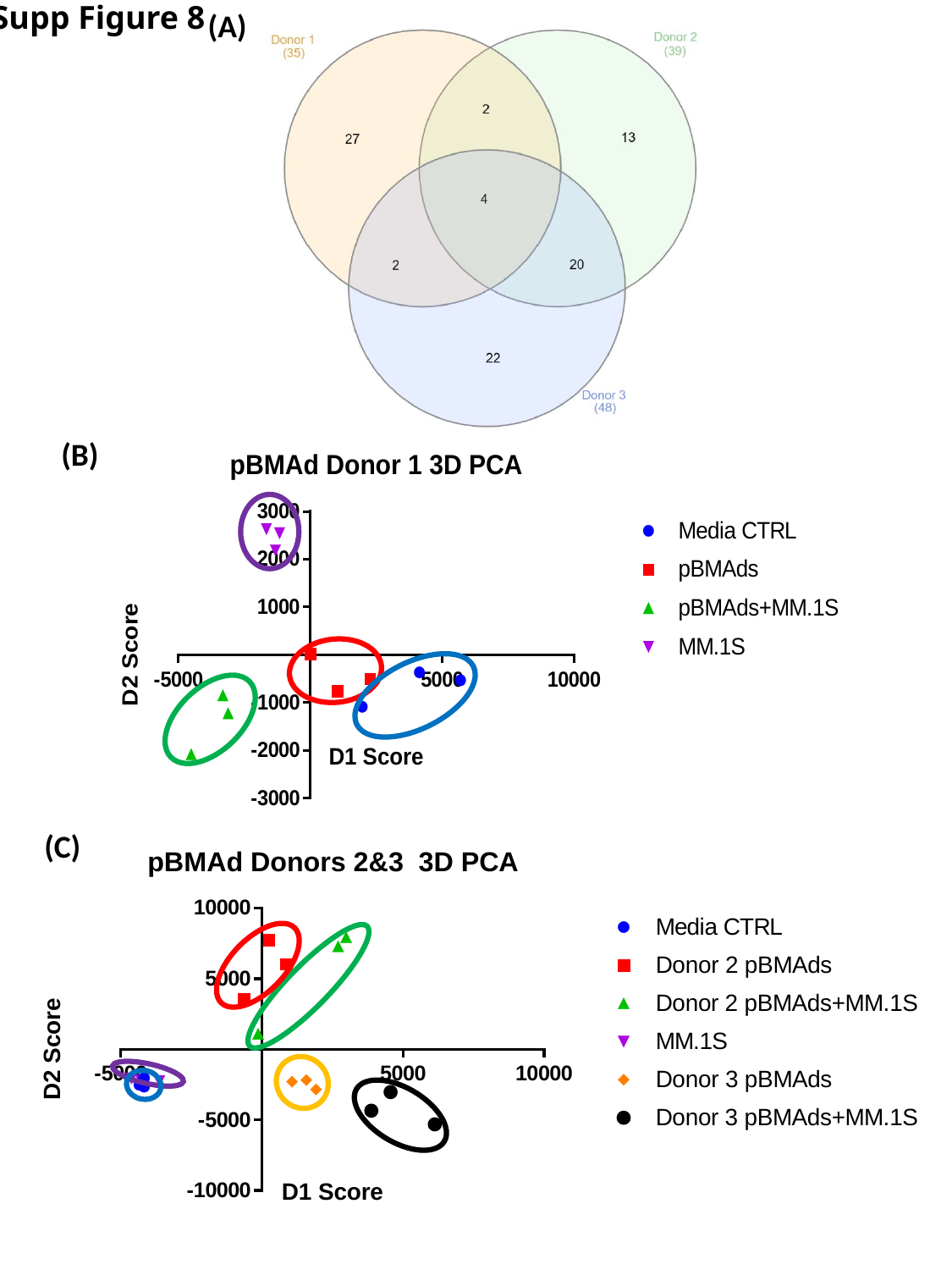

(A)
Supp Figure 8
(B)
(C)

## Slide 11
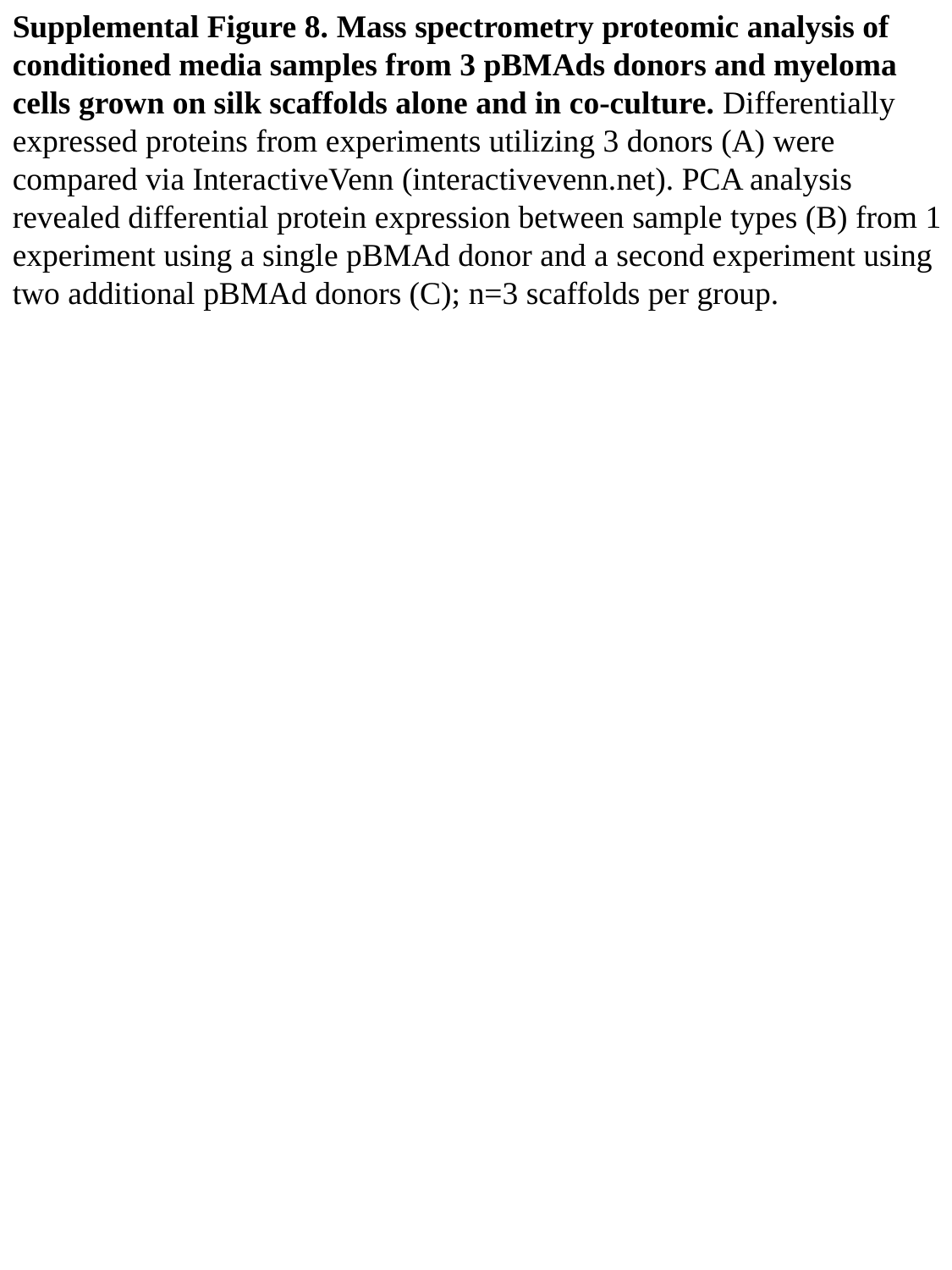

Supplemental Figure 8. Mass spectrometry proteomic analysis of conditioned media samples from 3 pBMAds donors and myeloma cells grown on silk scaffolds alone and in co-culture. Differentially expressed proteins from experiments utilizing 3 donors (A) were compared via InteractiveVenn (interactivevenn.net). PCA analysis revealed differential protein expression between sample types (B) from 1 experiment using a single pBMAd donor and a second experiment using two additional pBMAd donors (C); n=3 scaffolds per group.
